# Comparative analysis of global transcriptome, proteome and acetylome in house dust mite‐induced murine allergic asthma model

**DOI:** 10.1002/ctm2.590

**Published:** 2021-11-06

**Authors:** Yahui Liu, Qianru Huang, Juan Du, Chunrong Huang, Dan Li, Xueyu Dai, Rui Liang, Bin Li, Guochao Shi

**Affiliations:** ^1^ Department of Respiratory and Critical Care Medicine Ruijin Hospital Shanghai Jiao Tong University School of Medicine Shanghai China; ^2^ Department of Immunology and Microbiology Shanghai Institute of Immunology Shanghai Jiao Tong University School of Medicine Shanghai China

Dear Editor,

Protein lysine‐site acetylation (Kac) is of great importance for various cellular processes, which indicates a reversible post‐translational modification of proteins. Previous studies have demonstrated that protein acetylation was involved in the pathogenesis of asthma.^1^ It has been shown that lysine acetyltransferase (KAT) activity is increased in asthma, while lysine deacetylase (KDAC) activity is reduced.^1^ With the development of high‐throughput omics techniques, crucial processes and factors that play important roles in asthma have recently been identified. However, systematic understanding of acetylome in asthma is hampered by several major deficiencies.

In order to detect the changes of acetylated protein in allergic asthma, we used a house dust mite (HDM)‐treated murine allergic asthma model, and we found that there were differences between the phosphate buffer saline (PBS)‐ and HDM‐treated groups via immunoblotted with pan‐acetyl‐lysine antibody (Ac‐lys)^2^ (Figure 1A,B). We isolated three lung tissues from PBS‐ and HDM‐treated mice to further clarify the acetylation mechanism and determine the acetylome via liquid chromatography tandem mass spectrometry (LC‐MS/MS) (Figure 1C and Table S1). Figure S1 showed the MS data validations of acetylome and indicated the mass accuracy. Principal component analysis demonstrated that the control and HDM‐treated lung can be classified into two clusters (Figure 1D). Pearson's correlation coefficient and relative standard deviation indicated acceptable accuracy and reproducibility (Figure S1). Proteome analysis was also performed, serving as background control for quantifying alterations of acetylation (Figure S2 and Table S2). After comparative acetylome analysis, we identified 5132 Kac sites from 1860 proteins and quantified 3176 sites from 1197 proteins from these in the lung (Figure S1A). Compared to the control group, 34 sites from 31 proteins were upregulated, and 99 sites from 93 proteins were downregulated on HDM treatment (Figure 1E). One thousand three hundred eighty‐three acetylated proteins and 3457 Kac sites were shared in both groups (Figure 1F,G). The five proteins with most Kac sites were myosin‐9 (Q8VDD5, 44 Kac sites), spectrin alpha chain (P16546, 42 Kac sites), myosin‐6 (Q02566, 33 Kac sites), filamin‐A (Q8BTM8, 32 Kac sites) and fatty acid synthase (P19096, 31 Kac sites) (Table S1).

**FIGURE 1:**
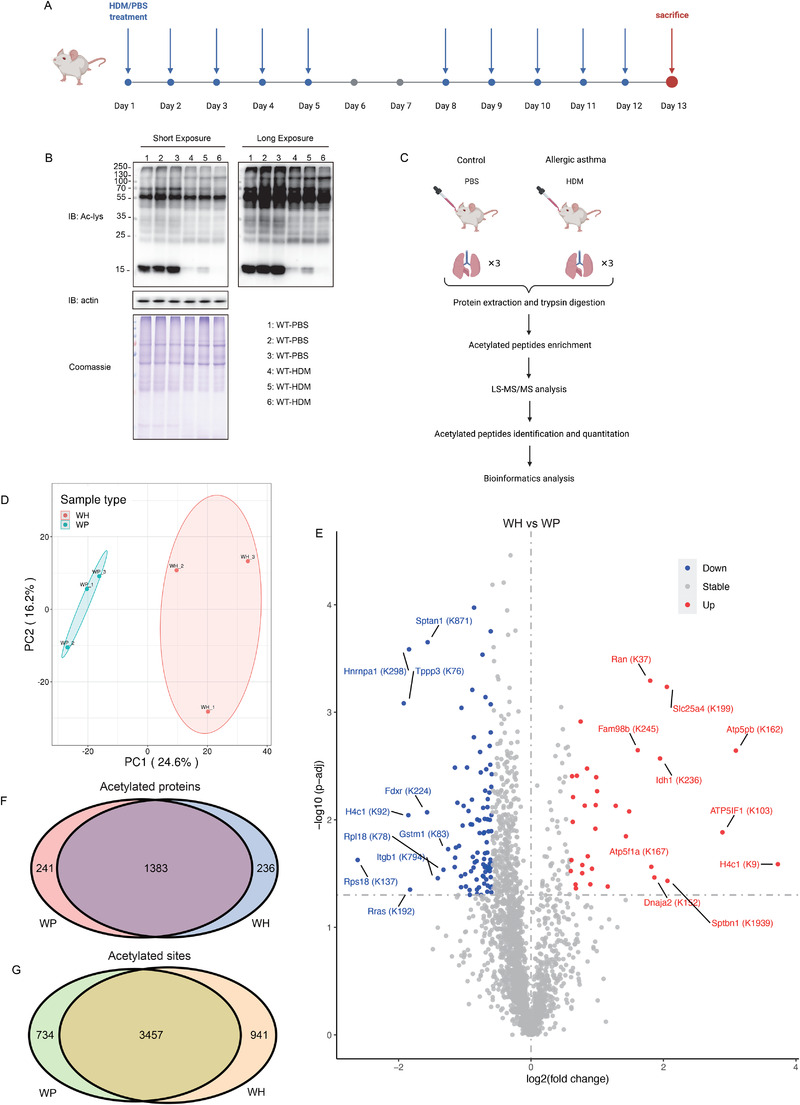
Proteome‐wide identification of lysine acetylation proteins and sites in HDM‐induced murine allergic asthma model. (A) Schematic diagram of allergic asthma model. HDM, house dust mite. PBS, phosphate buffer saline. (B) Detection of lysine acetylation in mouse lungs of PBS‐treated (*n* = 3) and HDM‐challenged (*n* = 3) wild‐type mice using pan‐acetyl‐lysine antibody (Ac‐lys). Equal loading was verified using β‐actin western blot and Coomassie blue staining. WT, wild‐type. IB, immunoblot. (C) Experimental flow chart. Six 6‐ to 8‐week‐old C57BL/6 female mice were divided into two groups: PBS‐treated control group (*n* = 3) and HDM‐treated group (*n* = 3). All mice were sacrificed by overdose of pentobarbital 24 h after the last challenge, and lung tissues were isolated for liquid chromatography tandem mass spectrometry (LC‐MS/MS). (D) Principal component analysis (PCA) of acetylome data from lung tissues. (E) Volcano plot of differentially expressed acetylated protein lysine sites between control and HDM‐treated group. Venn diagram of acetylated proteins (F) and lysine sites (G) from PBS‐treated and HDM‐treated group mouse lung tissues. WH, HDM‐treated WT mice. WP, PBS‐treated WT mice.

Previously, transcriptome analysis has demonstrated that various important processes were changed in HDM‐treated murine lung tissues. However, the murine strains and modelling methods used in literature reports are different from each other^3^, ^4^, ^5^, ^6^, ^7^, ^8^, ^9^, ^10^ (Table S3). Therefore, in order to comprehensively analyze the molecular regulation mechanism during allergic asthma and obtain more accurate results, we also performed RNA sequence analysis of the whole lung of the same murine allergic asthma model (Figure S4‐S6, Table S4).

Then, we systematically compared the transcriptome and proteome data of lung tissues and found that 322 genes showed significant changes in both mRNA and protein expression. Of these, 204 gene products were upregulated, and 118 were downregulated in both datasets (Figure 2A). Although limited gene products were shared between the transcriptome and proteome analyses, Gene Ontology (GO) and Kyoto Encyclopedia of Genes and Genomes (KEGG) analyses disclosed a large overlap of biological processes that were critical for immune response and inflammatory response (Figure S7). Additionally, GO analysis using lung proteomic data revealed a regulatory network of mouse lung tissues on HDM treatment which included neutrophil degranulation, actin cytoskeleton organization, regulation of cell adhesion, hemostasis, inflammatory response, cell‐substrate adhesion, actomyosin structure organization and other clusters (Figure S8). As is shown in Figure 2B, these processes showed significant enrichment in both transcriptome and proteome datasets. These data showed that there was a significant correlation between transcriptome and proteome levels in allergic asthma.

**FIGURE 2:**
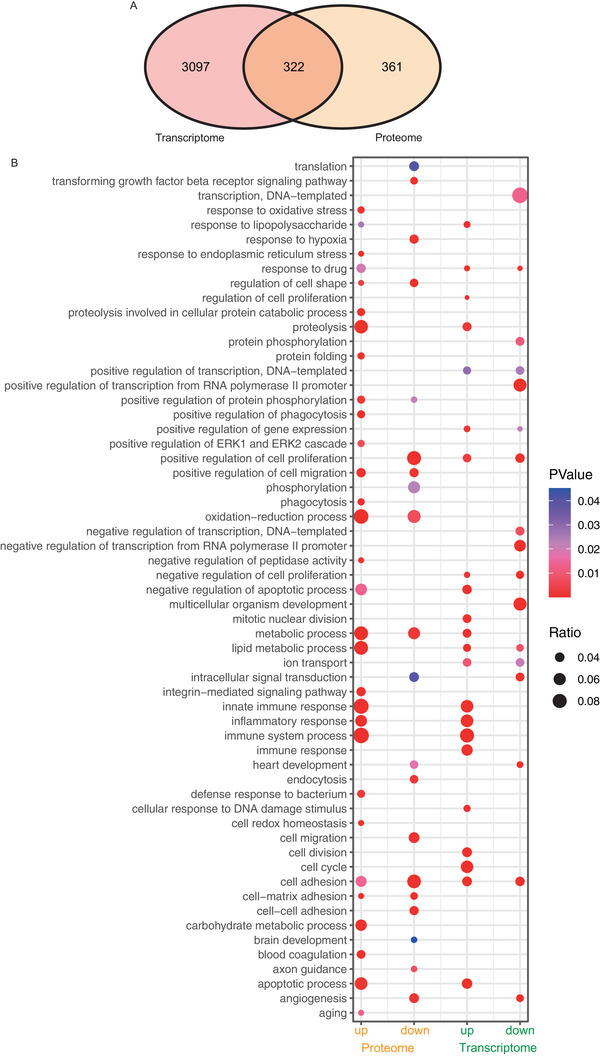
Correlation analyses between transcriptome and proteome data from mice lung tissues. (A) Venn diagram of RNA‐seq data and proteome data. (B) Comparative Kyoto Encyclopedia of Genes and Genomes (KEGG) analyses of transcriptome and proteome data. The KEGG analyses were performed using upregulated mRNAs, downregulated mRNAs, upregulated proteins and downregulated proteins. The correlation between two datasets is demonstrated. Gene ratios are indicated by circle sizes, and when *p_adj_* < 0.05, a significant change in the KEGG pathway was recognized.

To explore the effect of protein Kac on HDM challenge, we quantified the acetylated proteins and sites in the lung. Compared to the control group, proteins or sites with *p* < 0.05 and fold changes either greater than 1.5 or less than 1/1.5 were considered significantly changed. The GO analysis, using all significantly changed acetylated proteins, showed that ATP metabolic process, substrate adhesion‐dependent cell spreading, protein transport and other biological processes are regulated in lung tissues (Figure S9A). The biological processes enriched by the upregulated and downregulated acetylated proteins were in the Figure S9. In addition, the cellular component and molecular function via GO analysis were in Figure S10. KEGG analyses showed that there were huge differences in the biological processes of differentially expressed acetylated proteins between the two groups (Figure S9D). To detect the properties and features of acetylated sites or peptides, Motif‐X program was used for motif analysis. The results showed that 10 consensus sequence motifs were enriched, including K^ac^H, K^ac^S, K^ac^Y, GK^ac^T, K^ac^N, AK^ac^T, K^ac^F, K^ac^R, K^ac^T, K^ac^K, TK^ac^V, TK^ac^, GK^ac^G, K^ac^V and K^ac^W (K^ac^ indicates the acetylated lysine [Table S5 and Figure S9E]).

Then, we sorted all quantifiable protein Kac sites into four quantitative groups (down‐regulated by 0.5 times or less as Q1, down‐regulated by 0.5–0.667 times as Q2, up‐regulated by 1.5–2 times as Q3 and up‐regulated by more than two times as Q4) for the following GO and KEGG analysis. There were 21, 78, 20 and 14 proteins Kac sites in Q1, Q2, Q3 and Q4, respectively (Figure 3A and Table S6). Proteins in Q1 mainly localized in small ribosomal subunit, intercalated disc, cell‐cell contact zone and filopodium via cellular component analysis as described in Figure 3B. Proteins in Q3 mainly localized in microtubule cytoskeleton and cytoskeletal part, while proteins in Q4 in mitochondrial inner membrane (Figure 3B). The biological process enrichment of acetylation was shown in Figure 3C, and Figure 3D showed the molecular function analysis. KEGG‐based pathway analysis showed that Q1 proteins are enriched in ribosome, phagosome, axon guidance and Rap1 signaling pathway; Q2 proteins in drug metabolism, adrenergic signaling in cardiomyocytes, apoptosis and folate biosynthesis; Q3 proteins in Kaposi sarcoma‐associated herpesvirus infection, oocyte meiosis and insulin signaling pathway; and Q4 proteins in amino sugar and nucleotide sugar metabolism, human T‐cell leukemia virus 1 infection, Parkinson disease and Huntington disease (Figure 3E).

**FIGURE 3:**
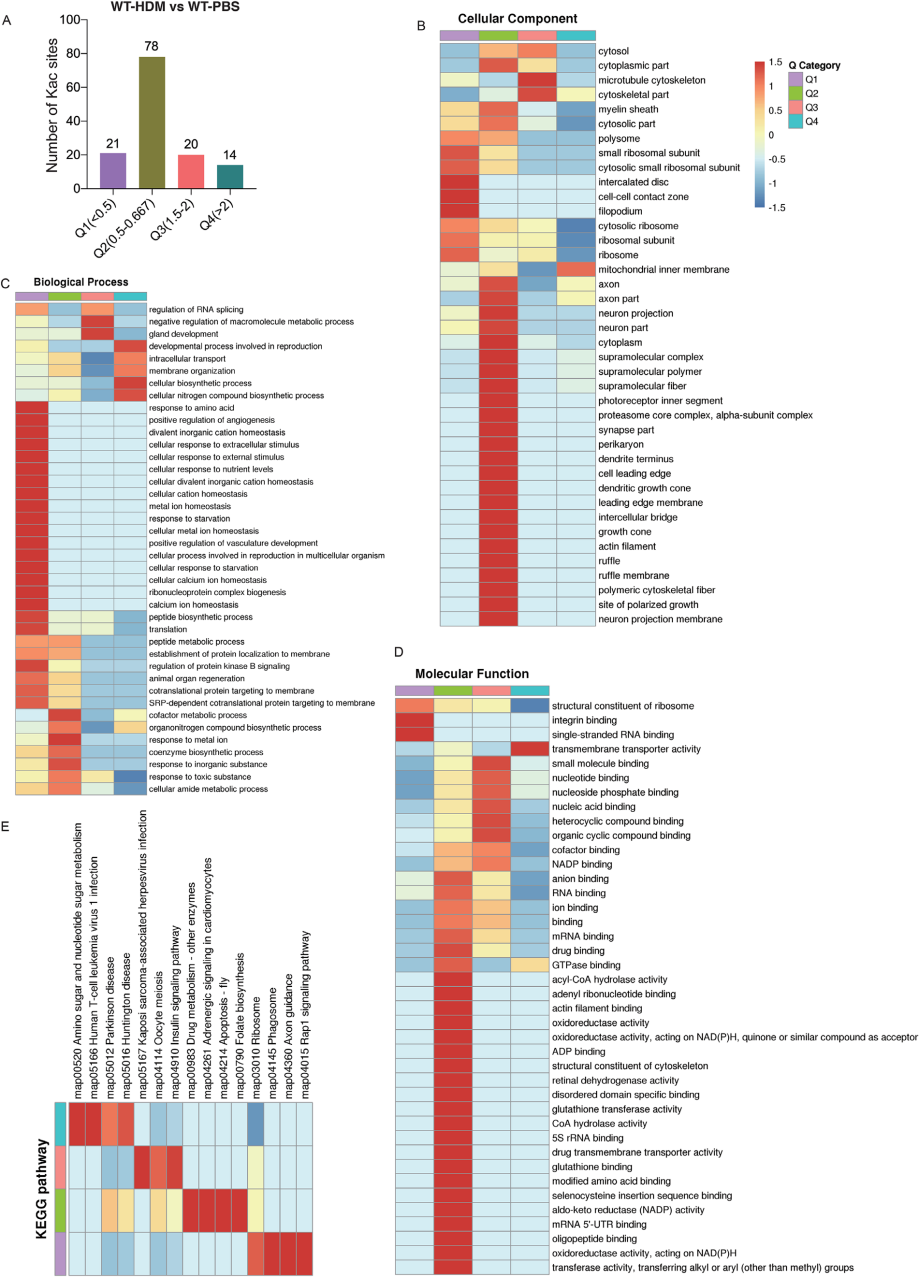
Functional enrichment‐based clustering analysis for the quantified acetylome. (A) The numbers of proteins Kac sites in each cluster. (B) Cellular component analysis. (C) Biological process analysis. (D) Molecular function analysis. (E) Kyoto Encyclopedia of Genes and Genomes (KEGG)‐based functional enrichment analysis for the quantified acetylome.

Additionally, we executed a protein–protein interactions (PPI) network analysis to delineate the PPI with differentially acetylated proteins, and 124 proteins were employed in the reactome network (Figure 4A). The acetylated proteins associated with HDM treatment could fall into several groups: metabolic process group (purine ribonucleotide metabolic process and nucleotide metabolic process), cellular component organization group (actin filament organization and cellular component organization), mitochondrial transport group, regulation of endothelial cell proliferation group, positive regulation of cell‐substrate adhesion group and cellular response to epidermal growth factor stimulus group (Figure 4A). These networks indicate that HDM treatment modulates the acetylation status of proteins in lung tissues, and those proteins are involved in organelle localization, metabolic process and cellular component organization and other biological processes in the murine allergic asthma model.

**FIGURE 4:**
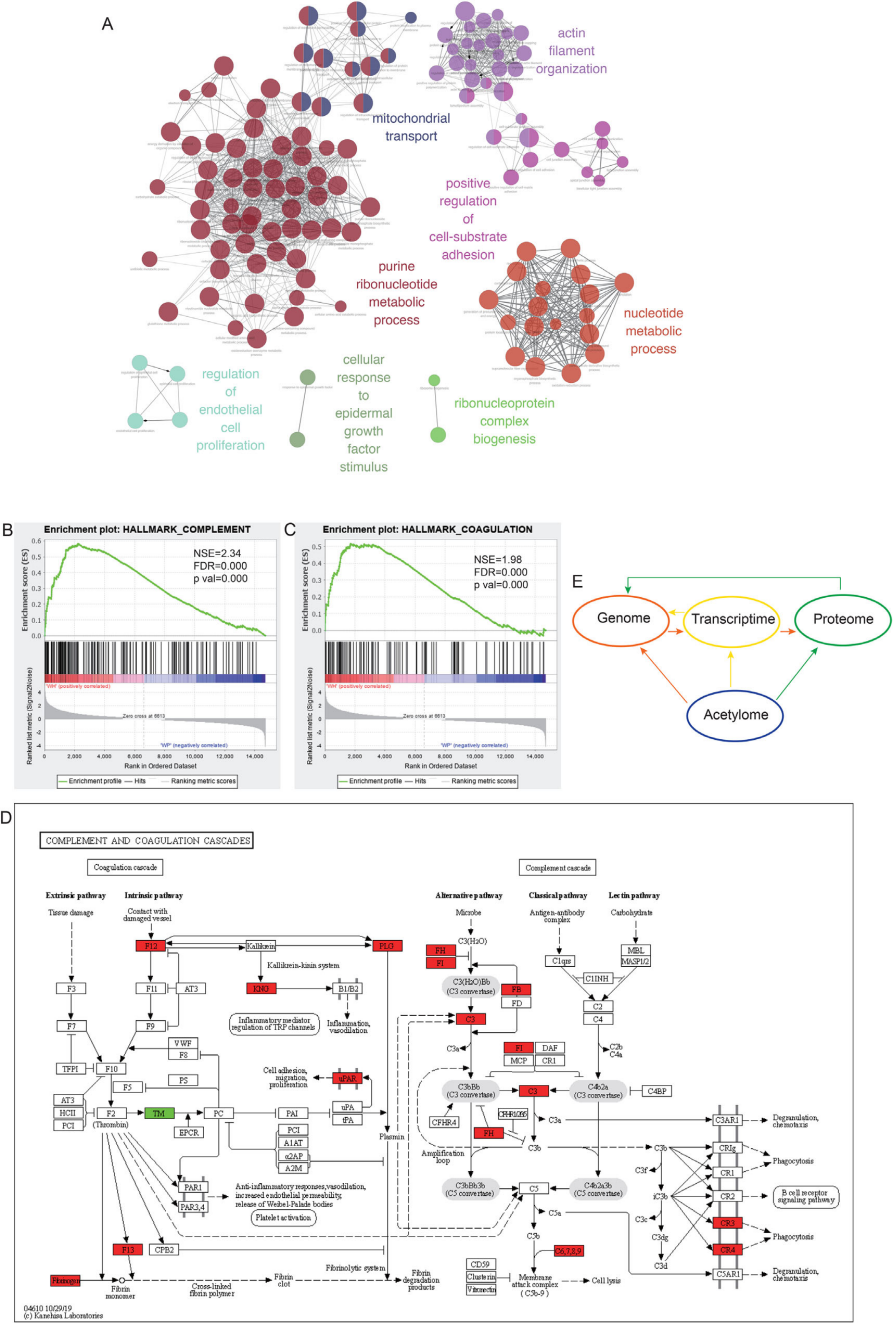
(A) Acetylome Gene Ontology (GO) analyses of house dust mite (HDM)‐treated mouse lungs. The GO reactome analysis was performed using differentially expressed acetylated proteins in phosphate buffer saline (PBS)‐treated and HDM‐treated mouse lung tissues. Gene Set Enrichment Analysis (GSEA) of gene sets associated with complement (B) and coagulation (C). (D) Kyoto Encyclopedia of Genes and Genomes (KEGG) pathway engine analysis of the complement and coagulation cascade pathway for PBS‐treated and HDM‐treated mouse lung tissues. (E) Schematic diagram of acetylation regulating gene expression, transcriptome and acetylome.

With the help of advanced bioinformatic analysis, we compared transcriptome, proteome and acetylome data, which revealed critical biological process and pathway related to HDM‐induced allergic airway inflammation (Figure S11). Both the complement system and coagulation cascade participate in the pathogenesis of asthma. In our data, upregulated genes of HDM‐treated mouse lung tissues were enriched in complement‐ and coagulation‐related genes (Figure 4B,C). Figure 4D showed the results of mapping the screen hit data for the proteome data onto the complement and coagulation cascade KEGG pathway and indicated the proteins in this pathway that were screen hits. However, there is a little information about complement and coagulation cascade in acetylome data (Table S1). The relationship between acetylome and transcriptome or proteome is complicated (Figure 4E). Histone acetylation to regulate the transcriptional activity is of great importance. Studies in recent years have confirmed that RNA can be acetylated, indicating that acetylation maybe a kind of post‐transcriptional modification. RNA binding to the catalytic domain of KATs can stimulate the enzyme activity of KATs, thereby affecting gene expression. Acetylation modification participates in biological processes by affecting various functions of proteins. Transcriptional factors (TFs) can also regulate gene expression through acetylation, and a variety of KATs themselves act as transcription co‐activators. Therefore, we need more research to clarify the acetylation in asthma, such as the acetylation modification of non‐coding RNA, the effect of acetylation on the activity of TFs and the presence or changes of specific acetylated lysine sites and its role in asthma.

In summary, we employed RNA sequence technology, proteome and acetylome MS analyses to systematically determine the molecular modulation mechanisms of mouse lung tissues in response to HDM treatment. These findings highlighted the importance of incorporating different measurements of gene expression. Finally, our data are open to the vast number of scientific researchers. Combined with other approaches, we can have a deeper understanding of the pathogenesis of asthma.

## CONFLICT OF INTERESTS

The authors declare no competing interests.

## Supporting information

Supporting Information

Supporting Information

Supporting Information

Supporting Information

Supporting Information

Supporting Information

Supporting Information

Supporting Information

Supporting Information

Supporting Information

Supporting Information

Supporting Information

Supporting Information

Supporting Information

Supporting Information

Supporting Information

Supporting Information

Supporting Information
