# Supplementary material for: Comparative analysis of global transcriptome, proteome and acetylome in house dust mite‐induced murine allergic asthma model
Source: Clin Transl Med. 2021 Nov 6;11(11):e590. doi: 10.1002/ctm2.590 (PMC8571946; doi:10.1002/ctm2.590)
Supplement: Supplementary file 1 — Supporting Information [file CTM2-11-e590-s010.docx]

**Supplemental materials for**

**Comparative analysis of global transcriptome, proteome and acetylome in house dust mite-induced murine allergic asthma model**

Yahui Liu^1†^, Qianru Huang^2†^, Juan Du^1^^†^, Chunrong Huang^1^, Dan Li^2^, Xueyu Dai^2^, Rui Liang^2^, Bin Li^2*^, Guochao Shi^1*^

**Materials and methods**

**1. Reagents and antibodies**

The following antibodies were used: acetylated lysine antibody (9441, Cell Signaling Technology (CST)), mouse anti-β-actin (60008, Proteintech, 1:10,000 for western blot), β-tubulin rabbit monoclonal antibody (2128, CST, 1:1,000 for western blot), IL-33 antibody (AF3626, R&D systems, 0.4 µg/mL for western blot), HDAC4 (7628, CST, 1:2,000 for western blot), HDAC5 (20458, CST, 1:1,000 for western blot), HDAC6 (7612, CST, 1:1,000 for western blot), STAT3 (12640, CST, 1:1,000 for western blot), Ac-Histone H3 (sc-56616, Santa Cruz Biotechnology, 1:200 for western blot), Histone H3 (4499, CST, 1:2,000 for western blot), Histone H4 (13919, CST, 1:1,000 for western blot), Ac-Histone H4 (sc-377520, Santa Cruz Biotechnology, 1:1000 for western blot), Collagen I (ab270993, abcam, 1:1,000 for western blot), TIP60 (sc-166323, Santa Cruz Biotechnology, 1:1000 for western blot), goat anti-rabbit IgG HRP-linked antibody (7074, CST 7074, 1:3,000 for western blot), and goat anti-mouse IgG (peroxidase conjugated, (H+L), 31430, Invitrogen, 1:5000 for western blot). House dust mite (HDM) extract was purchased from Greer Laboratories (Boston, Massachusetts, USA). Proteinase inhibitor cocktail (P8340) was purchased from Sigma-Aldrich (Darmstadt, Germany).

**2. Mice**

All animal experiments were performed in accordance with the National Institutes of Health Guide for the Care and Use of Laboratory Animals and with the approval of the Institutional Animal Care and Use Committee in Institute Pasteur of Shanghai. All mice were maintained under specific pathogen-free conditions.

Female 6- to 8- week-old C57BL/6 mice were used for experiments. For the allergic asthma model, mice were challenged for five consecutive days in two weeks with intranasal administration of 35 μL (0.7 mg/mL phosphate buffered saline (PBS)) of whole HDM protein extract. Control animals received only PBS. All mice were sacrificed by overdose of pentobarbital 24 hours after the last challenge.

**3. Western blot**

Lung tissues of PBS-treated (n = 3) and HDM-challenged mice (n = 3) were homogenized in lysis buffer (50 mM Tris-HCl, PH 7.4, 150 mM NaCl, 0.5 mM EDAT, 1mM DTT, 1% Triton X-100, and 0.5% sodium deoxycholate) which was supplemented with 1% proteinase inhibitor cocktail, 1 mM PMSF, 1 mM NaF, and 1 mM Na_3_VO_4_, and centrifuged at 12,000 rpm for 15 min at 4℃. The supernatant containing the tissue lysates was separated by SDS-PAGE and analyzed by immunoblotting. Proteins were visualized using enhanced chemiluminescence (WBKLS0500, Millipore).

**4. Acetylome analysis**

**4.1 Protein extraction**: The sample of PBS-treated (n = 3) and HDM-challenged mice (n = 3) was grinded by liquid nitrogen into cell powder and then transferred to a 5-mL centrifuge tube. After that, four volumes of lysis buffer (8 M urea, 1% Protease Inhibitor Cocktail, 3 μM TSA and 50 mM NAM) was added to the cell powder, followed by sonication three times on ice using a high intensity ultrasonic processor (Scientz). The remaining debris was removed by centrifugation at 12,000 g at 4 °C for 10 min. Finally, the supernatant was collected and the protein concentration was determined with Bradford Protein Assay Kit according to the manufacturer’s instructions.

**4.2 Trypsin digestion**: The protein solution was reduced with 5 mM dithiothreitol for 30 minutes at 56°C and alkylated with 11 mM iodoacetamide for 15 min at room temperature in darkness. The sample was then diluted by adding 100 mM TEAB to urea concentration less than 2M. Finally, trypsin was added at 1:50 trypsin-to-protein mass ratio for the first digestion overnight and 1:100 trypsin-to-protein mass ratio for a second 4 h-digestion.

**4.3 Acetylated peptides enrichment and LC-MS/MS analysis**: The samples were reconstituted in 1.4 mL of precooled IAP Buffer, added pretreated Anti-Ac-K antibody beads (PTMScan Acetyl-Lysine Motif (Ac-K) Kit, Cell Signaling Technology), then incubated at 4 ⁰C for 1.5 h, centrifuged at 2,000 ×g for 30s, then discarded the supernatant. Anti-Ac-K antibody beads were washed with 1mL precooled IAP Buffer for 3 times, than washed with precooled water for 3 times. Forty μL 0.15% TFA was added to the washed beads, incubated for 10 min at room temperature, then added 0.15% TFA again, centrifuged at 2,000 ×g for 30s, the supernatant was desalted by C18 STAGE Tips.

The tryptic peptides were dissolved in 0.1% formic acid (solvent A), directly loaded onto a home-made reversed-phase analytical column (15-cm length, 75 μm i.d.). The gradient was comprised of an increase from 6% to 23% solvent B (0.1% formic acid in 98% acetonitrile) over 26 min, 23% to 35% in 8 min and climbing to 80% in 3 min then holding at 80% for the last 3 min, all at a constant flow rate of 400 nL/min on an EASY-nLC 1000 UPLC system. The peptides were subjected to NSI source followed by tandem mass spectrometry (MS/MS) in Q ExactiveTM Plus (Thermo) coupled online to the UPLC. The electrospray voltage applied was 2.0 kV. The m/z scan range was 350 to 1800 for full scan, and intact peptides were detected in the Orbitrap at a resolution of 70,000. Peptides were then selected for MS/MS using NCE setting as 28 and the fragments were detected in the Orbitrap at a resolution of 17,500. A data-dependent procedure that alternated between one MS scan followed by 20 MS/MS scans with 15.0s dynamic exclusion. Automatic gain control (AGC) was set at 5E4. Fixed first mass was set as 100 m/z.

**4.4 Database search**

The resulting LC-MS/MS data were processed using MaxQuant search engine (v.1.5.2.8). Tandem mass spectra were searched against uniprot database concatenated with reverse decoy database. Trypsin/P was specified as cleavage enzyme allowing up to 4 missing cleavages. The mass tolerance for precursor ions was set as 20 ppm in First search and 5 ppm in Main search, and the mass tolerance for fragment ions was set as 0.02 Da. Carbamidomethyl on Cys was specified as fixed modification and acetylation modification and oxidation on Met were specified as variable modifications. FDR was adjusted to < 1% and minimum score for modified peptides was set > 40.

**4.5 bioinformatic analysis**

Enrichment of Gene Ontology analysis: Proteins were classified by GO annotation into three categories: biological process, cellular compartment and molecular function. For each category, a two-tailed Fisher’s exact test was employed to test the enrichment of the differentially expressed protein against all identified proteins. The GO with a corrected *p value* < 0.05 is considered significant.

Enrichment of pathway analysis: Encyclopedia of Genes and Genomes (KEGG) database was used to identify enriched pathways by a two-tailed Fisher’s exact test to test the enrichment of the differentially expressed protein against all identified proteins. The pathway with a corrected *p value* < 0.05 was considered significant. These pathways were classified into hierarchical categories according to the KEGG website.

Enrichment of protein domain analysis: For each category proteins, InterPro database was researched and a two-tailed *Fisher’s* exact test was employed to test the enrichment of the differentially expressed protein against all identified proteins. Protein domains with a corrected *p value* < 0.05 were considered significant.

For further hierarchical clustering based on differentially expressed protein functional classification. We first collated all the categories obtained after enrichment along with their *p values*, and then filtered for those categories which were at least enriched in one of the clusters with *p value* <0.05. This filtered *p value* matrix was transformed by the function x = −log10 (*p value*). Finally, these x values were z-transformed for each functional category. These z scores were then clustered by one-way hierarchical clustering (Euclidean distance, average linkage clustering) in Genesis. Cluster membership were visualized by a heat map using the “heatmap.2” function from the “gplots” R-package.

The protein–protein interaction (PPI) information of the studied proteins was retrieved from IntAct molecular interaction database (http://www.ebi.ac.uk/intact/) by their gene symbols or STRING software (http://string-db.org/). The results were downloaded in the XGMML format and imported into Cytoscape software (http://www.cytoscape.org/, version 3.2.1) to visualize and further analyze functional protein-protein interaction networks. Furthermore, the degree of each protein was calculated to evaluate the importance of the protein in the PPI network.

Kac motif analysis: The model of sequences constituting with amino acids in specific positions of modify-21-mers (10 amino acids upstream and downstream of the Kac site) in all protein sequences were downloaded at www.uniprot.org and analyzed. Statistical significance for the enrichment of Kac motifs were calculated using Fisher test. In addition, all the database protein sequences were used as the background database parameter, and the other parameters remained as defaults.

**5. Proteome analysis:** Upon dissection, lung tissues from three PBS-treated and three HDM-treated mice were used for proteome analysis. The methods of protein extraction, trypsin digestion, LS-MS/MS analysis, database search and bioinformatic analysis for proteome were similar to those for acetylome. All LS-MS/MS analysis in this study was performed by Jingjie PTM Bio (China).

**6. RNA sequence**

**6.1 Sample collection and preparation for RNA sequence:** Total RNA was extracted from whole mouse lungs of PBS-treated (n = 3) and HDM-challenged mice (n = 3) using TRIzol as described previously ^1^. Snap-freeze the tissue to be extracted, and using a precooled mortar and pestle, grind the frozen tissue under liquid nitrogen.

RNA degradation and contamination were monitored on 1% agarose gels. RNA purity was checked using the NanoPhotometer® spectrophotometer (IMPLEN, CA, USA), and RNA concentration was measured using Qubit® RNA Assay Kit in Qubit® 2.0 Flurometer (Life Technologies, CA, USA). RNA integrity was assessed using the RNA Nano 6000 Assay Kit of the Bioanalyzer 2100 system (Agilent Technologies, CA, USA).

**6.2 Library preparation for transcriptome sequencing**: 3 μg RNA of each lung sample was used as input material for RNA sample preparations. Sequencing libraries were generated using NEBNext® UltraTM RNA Library Prep Kit for Illumina® (NEB, USA) following manufacturer’s recommendations and index codes were added to attribute sequences to each sample.

**6.3 Clustering and sequencing**: The clustering of index-coded samples was performed om a cBot Cluster Generation System using TruSeq PE Cluster Kit v3-cBot-HS (Illumia) according to the manufacturer’s instructions. After cluster generation, the library preparations were sequenced on an Illumina Hiseq platform and 125 bp/150 bp paired-end reads were generated.

**6.4 Data analysis:** Index of the reference genome was built using STAR and paired-end clean reads were aligned to the reference genome using STAR (v2.5.1b). STAR used the method of Maximal Mappable Prefix (MMP) which can generate a precise mapping result for junction reads. HTSeq v0.6.0 was used to count the reads numbers mapped to each gene. Differential expression analysis of two conditions/groups (two biological replicates per condition) was performed using the DESeq2 R package (1.10.1). DESeq2 provide statistical routines for determining differential expression in digital gene expression data using a model based on the negative binomial distribution. The resulting *p values* were adjusted using the *Benjamini* and *Hochberg’s* approach for controlling the false discovery rate. Genes with an adjusted *p value* <0.05 found by DESeq2 were assigned as differentially expressed. Gene Ontology (GO) enrichment analysis of differentially expressed genes was implemented by the clusterProfiler R package, in which gene length bias was corrected. GO terms with corrected *p value* less than 0.05 were considered significantly enriched by differential expressed genes. In addition, we used clusterProfiler R package to test the statistical enrichment of differential expression genes in KEGG pathways.

**7. Quantitative real-time PCR:** Total RNA was extracted from mouse lungs using TRIzol. RNA was quantified and complementary DNA was reverse transcribed with 1 μgRNA using the PrimeScript RT Reagent Kit (Takara, RR037A). PCR reactions were run in an ABI Prism 7500 Sequence Detection System. Quantification of relative mRNA expression was determined by the comparative CT method. Relative gene expression was determined via normalization to the housekeeping gene β-actin. Primers are as follows:

*β-actin* forward, GGCTGTATTCCCCTCCATCG;

*β-actin* reverse, CCAGTTGGTAACAATGCCATGT;

*Il13* forward, CGGCAGCATGGTATGGAGTGTG;

*Il13* reverse, GGAGGCTGGAGACCGTAGTGG;

*Il33* forward, CTGGCCTCACCATAAGAAAGGAGA;

*Il33* reverse, AGGGAGGCAGGAGACTGTGTTAAA;

*Tlr4* forward, ATGGCATGGCTTACACCACC;

*Tlr4* reverse, GAGGCCAATTTTGTCTCCACA;

*Hif1a* forward, ACCTTCATCGGAAACTCCAAAG;

*Hif1a* reverse, CTGTTAGGCTGGGAAAAGTTAGG;

*Esco2* forward, ATGGCAACTTGTACTCCAAGAAA;

*Esco2* reverse, GTTGGTGGGAGCAAGACTTGT;

*Muc5ac* forward, CAGGACTCTCTGAAATCGTACCA;

*Muc5ac* reverse, AAGGCTCGTACCACAGGGA;

*Il5* forward, CTCTGTTGACAAGCAATGAGACG;

*Il5* reward, TCTTCAGTATGTCTAGCCCCTG;

*Il6* forward, TAGTCCTTCCTACCCCAATTTCC ;

*Il6* reward, TTGGTCCTTAGCCACTCCTTC.

**8. Immunohistochemistry:** Immunohistochemistry was performed as described previously ^2^. Mouse lung tissue sections were incubated with primary antibody overnight at 4°C. Peroxidase-conjugated secondary antibody was then used for one hour at 37°C, followed by addition of the streptavidin-HRP conjugate and substrate chromogen mixture. Finally, all slides were counterstained with hematoxylin.

**Reference**

1. Donald C Rio, Manuel Ares Jr, Gregory J Hannon, et al. Purification of RNA using TRIzol (TRI reagent). Cold Spring Harb Protoc. 2010(6):pdb.prot5439. doi: 10.1101/pdb.prot5439.

2. Tian Liu, Yahui Liu, Marina Miller, et al. Autophagy plays a role in FSTL1-induced epithelial mesenchymal transition and airway remodeling in asthma. Am J Physiol Lung Cell Mol Physiol. 2017;313(1):L27-L40. doi: 10.1152/ajplung.00510.2016.

**Figure legend of supplemental materials**

**Figure S1**. (**A**) Survey of the acetylomes and Kac sites. (**B**) Identified peptide length distribution. (**C**) Peptide mass tolerance distribution. Pearson’s correlation analysis (**D**) and relative standard deviation (RSD) analysis (**E**) of acetylome data from PBS- and HDM-treated mouse lung tissues. WH, HDM-treated wild type (WT) mice. WP, PBS-treated WT mice.

**Figure S2**. (**A**) Survey of the proteome. (**B**) Identified peptide length distribution. (**C**) Identified protein mass distribution. (**D**) Protein sequence coverage distribution. (**E**) Protein mass and coverage distribution. (**F**) The number of peptides per protein distribution. (**G**) Principal component analysis (PCA) of proteome data from PBS- and HDM-treated mouse lung tissues. Pearson’s correlation analysis (**H**) and RSD analysis (**I**) of proteome data from PBS- and HDM-treated mouse lung tissues. WH, HDM-treated wild type (WT) mice. WP, PBS-treated WT mice.

**Figure S3. Verification of proteome and acetylome data.** (**A**) Nine proteins, namely HDAC4, STAT3, HDAC5, TIP60, Collagen I, HDAC6, IL33, Histone H3 and Histone H4 were detected by Western blot. The pan-acetylation levels of Histone H3 and Histone H4 were detected by Western blot. Tubulin was used as a control for protein quantification. (**B**) Hematoxylin and eosin (HE) staining, Periodic acid–Schiff (PAS) staining, immunohistochemistry staining (Collagen I, TIP60, IL33 and KAT1) of PBS-treated and HDM-treated mouse lung tissue sections.

**Figure S4**. (**A**) Schematic diagram of allergic asthma model. (**B**) Volcano plot of differentially expressed genes between PBS-treated and HDM-treated group mouse lung tissues. (**C**) Pathway and process enrichment analysis were performed and top 20 clusters were selected. Network of enriched terms was colored by cluster ID, where nodes that shared the same cluster ID were typically close to each other. WH, HDM-treated wild type (WT) mice. WP, PBS-treated WT mice.

**Figure S5. Verification of transcriptome data. * < 0.05, ** < 0.01, *** < 0.001.**

**Figure S6**. Heatmap of enriched terms across differentially expressed genes from the PBS-treated and HDM-treated mouse lung tissues, colored by *p values*.

**Figure S7**. GO analysis of biological process, cellular component, and molecular function of upregulated proteins (**A**), downregulated proteins (**B**), upregulated genes (**C**), and downregulated genes (**D**).

**Figure S8**. Pathway and process enrichment analysis using proteome data were performed and top 20 clusters were selected. (**A**) Network of enriched terms was colored by cluster ID, where nodes that shared the same cluster ID were typically close to each other; and by colored by *p value* (**B**), where terms containing more genes tend to have a more significant *p value*. (**C**) Network of enriched terms represented as pie charts, where pies are color-coded based on the identities of the gene lists.

**Figure S9**. (**A**) GO-based functional enrichment analysis for the quantified acetylome. The biological processes enriched by the upregulated (**B**) and downregulated (**C**) acetylated proteins. (**D**) The analysis of KEGG pathway of all the quantified acetylome. (**E**) Heat map of the amino acid compositions of the acetylated peptides.

**Figure S10**. The cellular component and molecular function via GO analysis.

**Figure S11**. GO analysis of acetylome, proteome and transcriptome data. Significantly enriched GO terms are shown, and the GO processes in all three datasets were shown.
