# Supplementary figures and images for: Comparative analysis of global transcriptome, proteome and acetylome in house dust mite‐induced murine allergic asthma model
Source: Clin Transl Med. 2021 Nov 6;11(11):e590. doi: 10.1002/ctm2.590 (PMC8571946; doi:10.1002/ctm2.590)

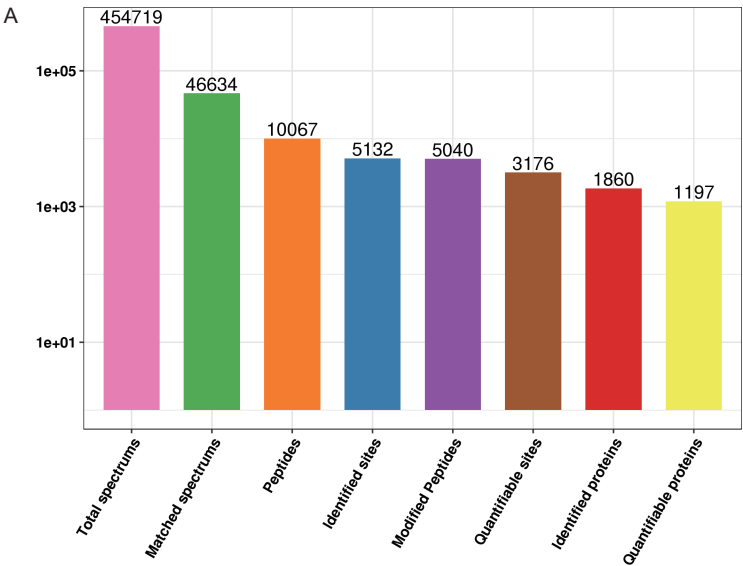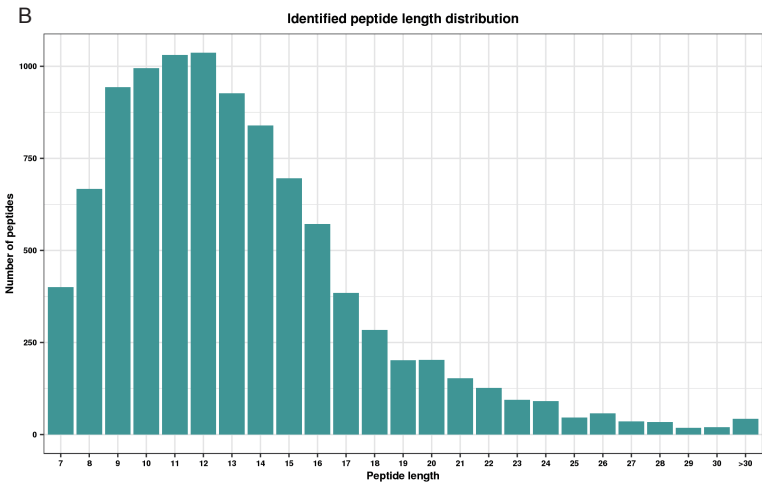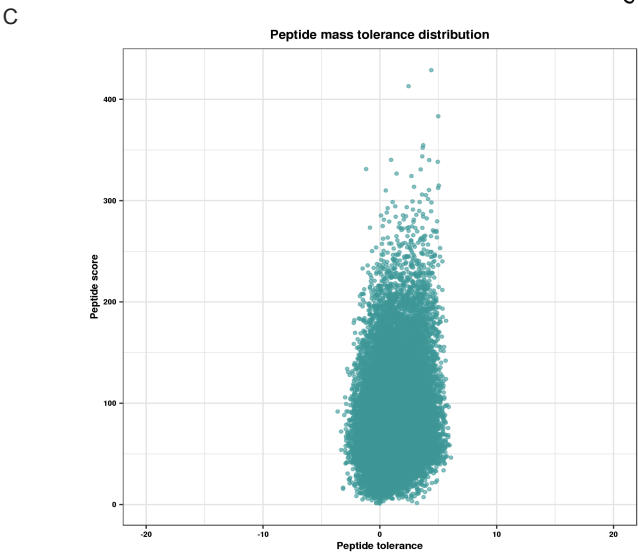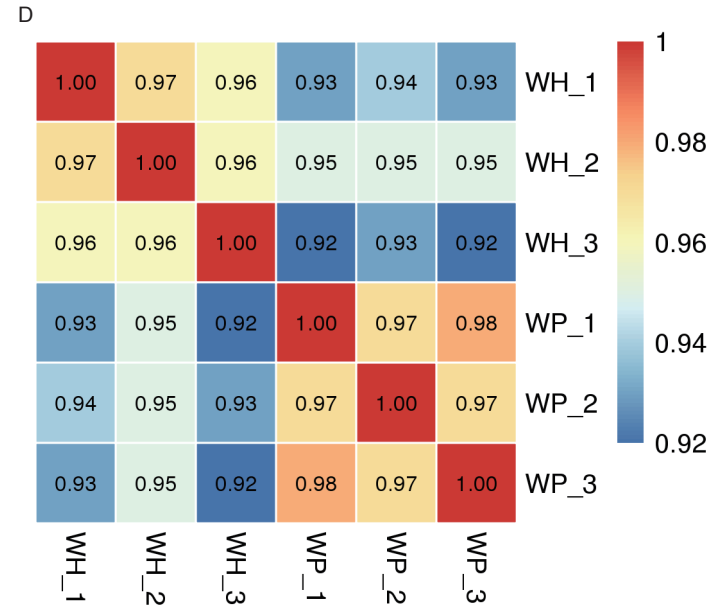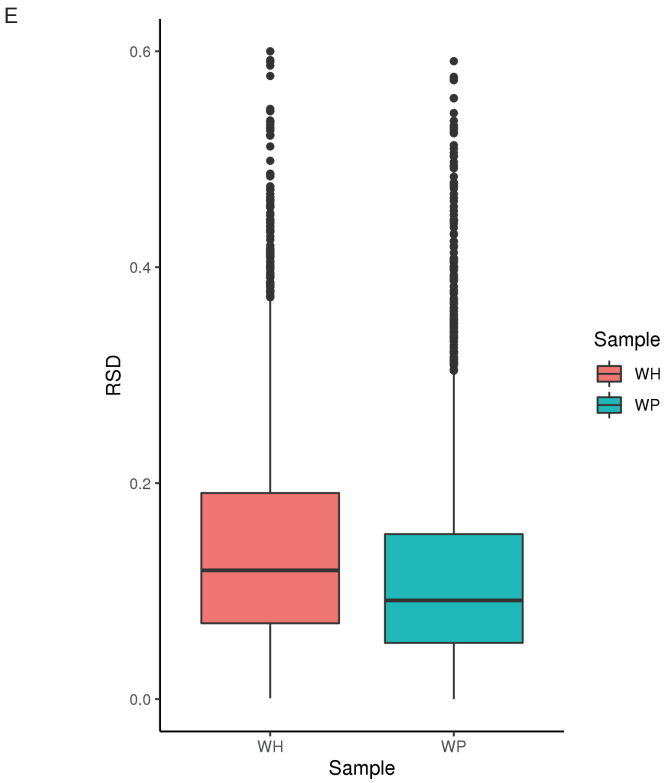

Supplement: Supplementary file 2 — Supporting Information [file CTM2-11-e590-s004.pdf]

A

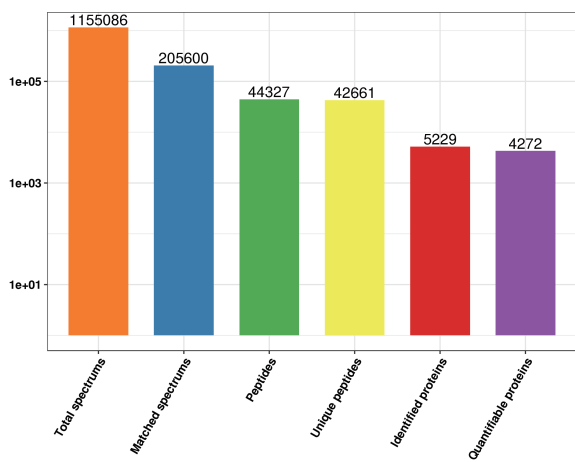

B

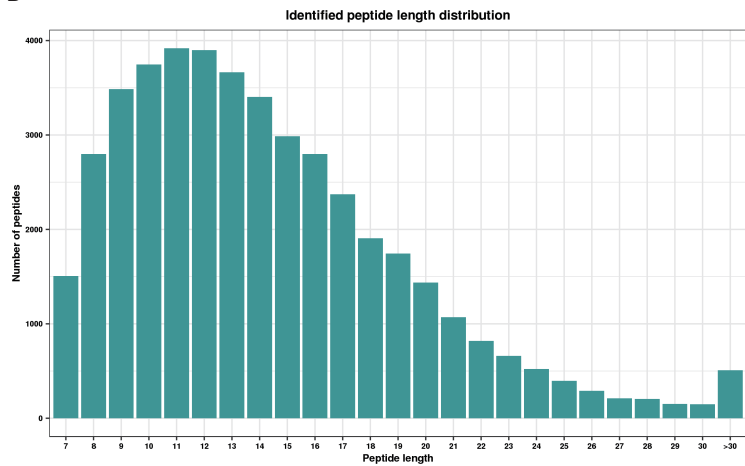

C

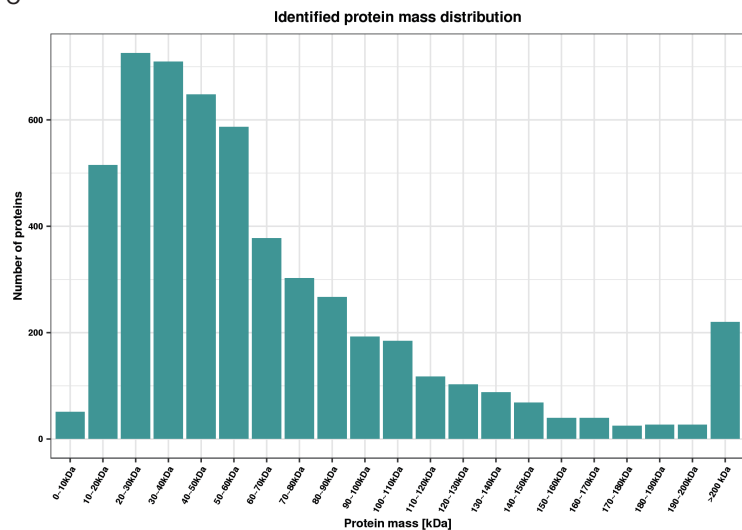

D

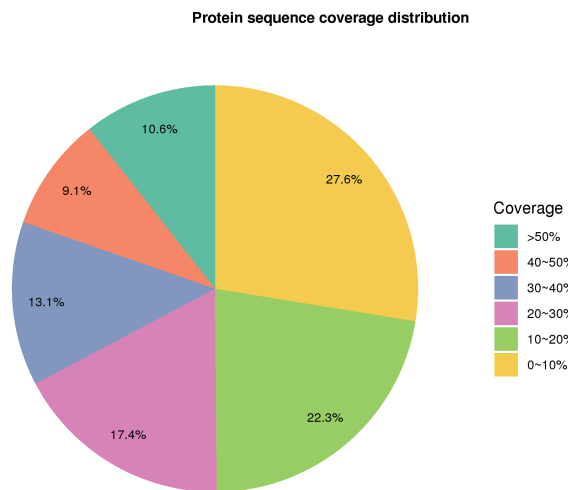

E

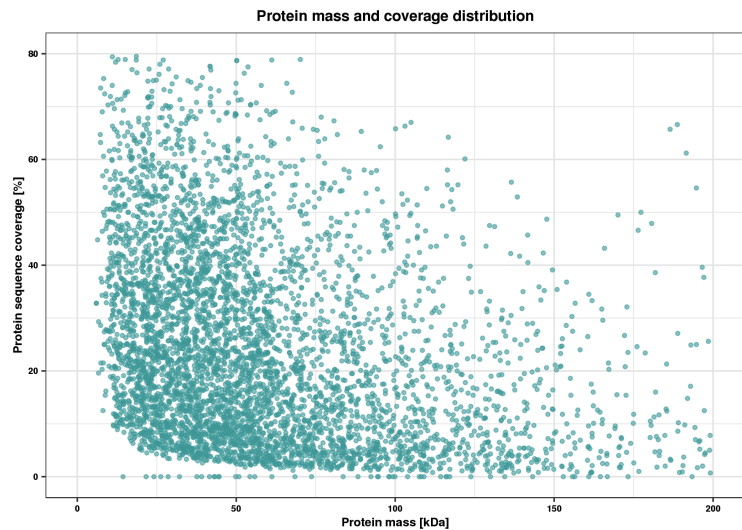

F

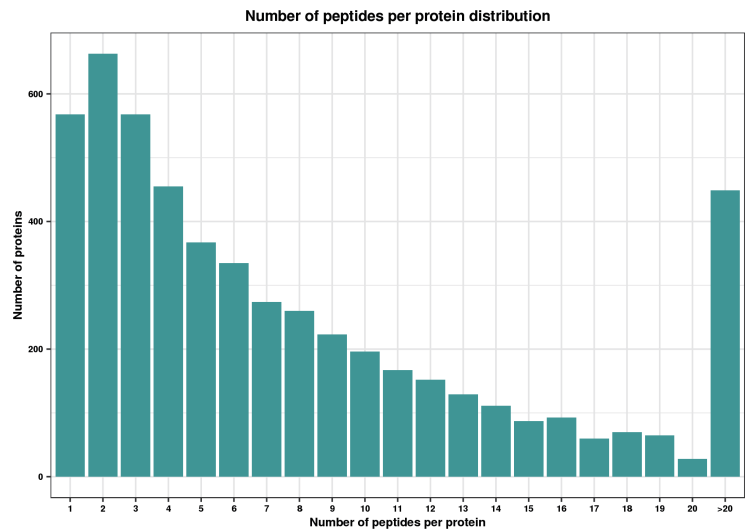

G

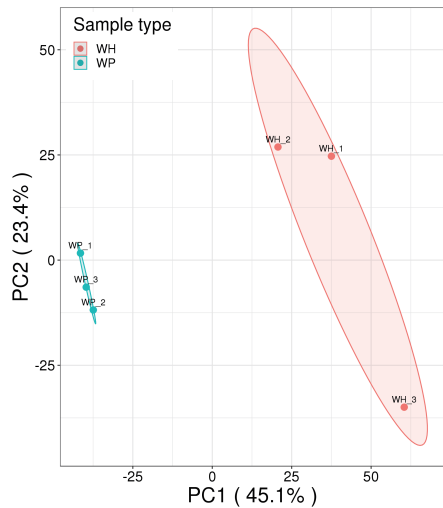

H

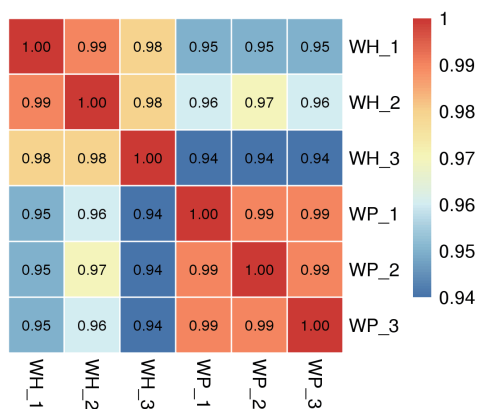

I

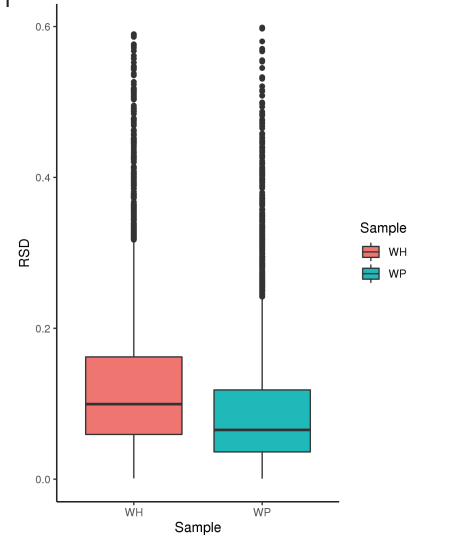

Supplement: Supplementary file 3 — Supporting Information [file CTM2-11-e590-s011.pdf]

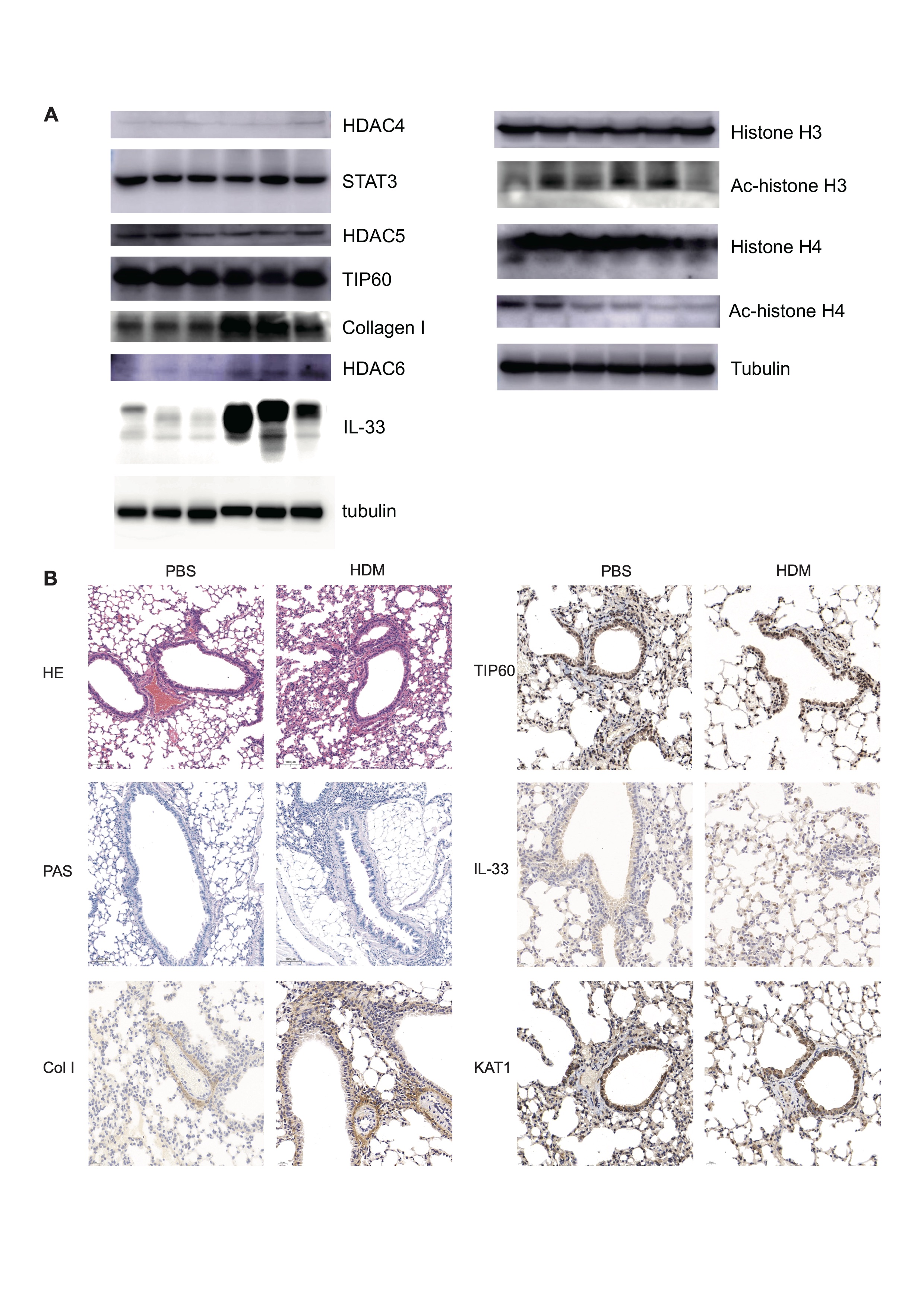

Supplement: Supplementary file 4 — Supporting Information [file CTM2-11-e590-s006.jpg]

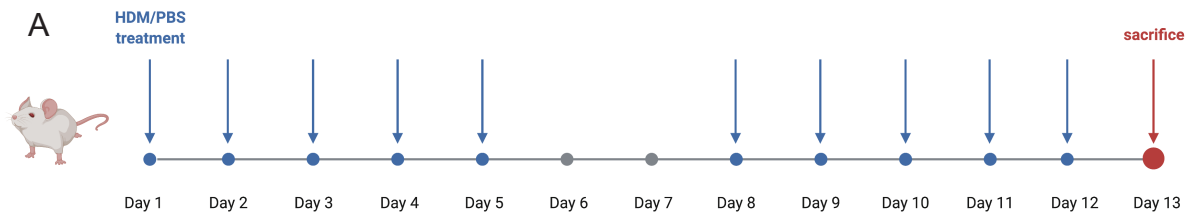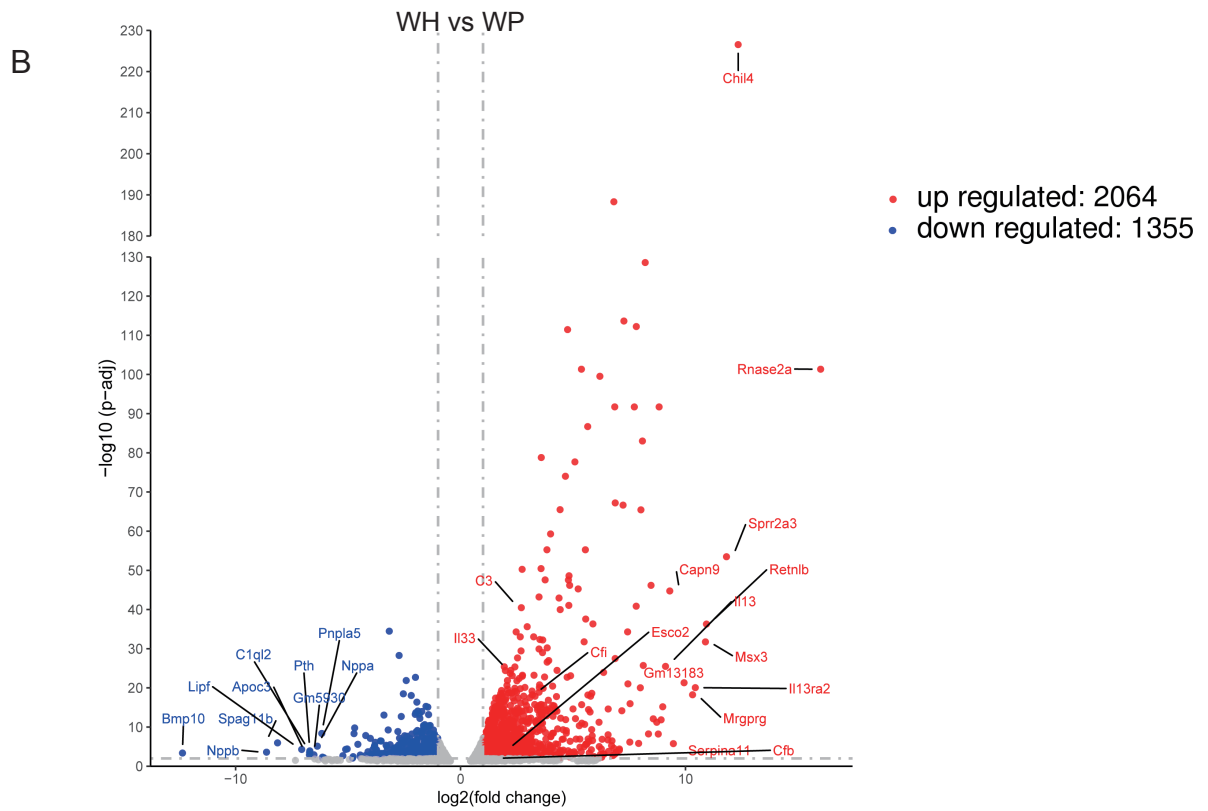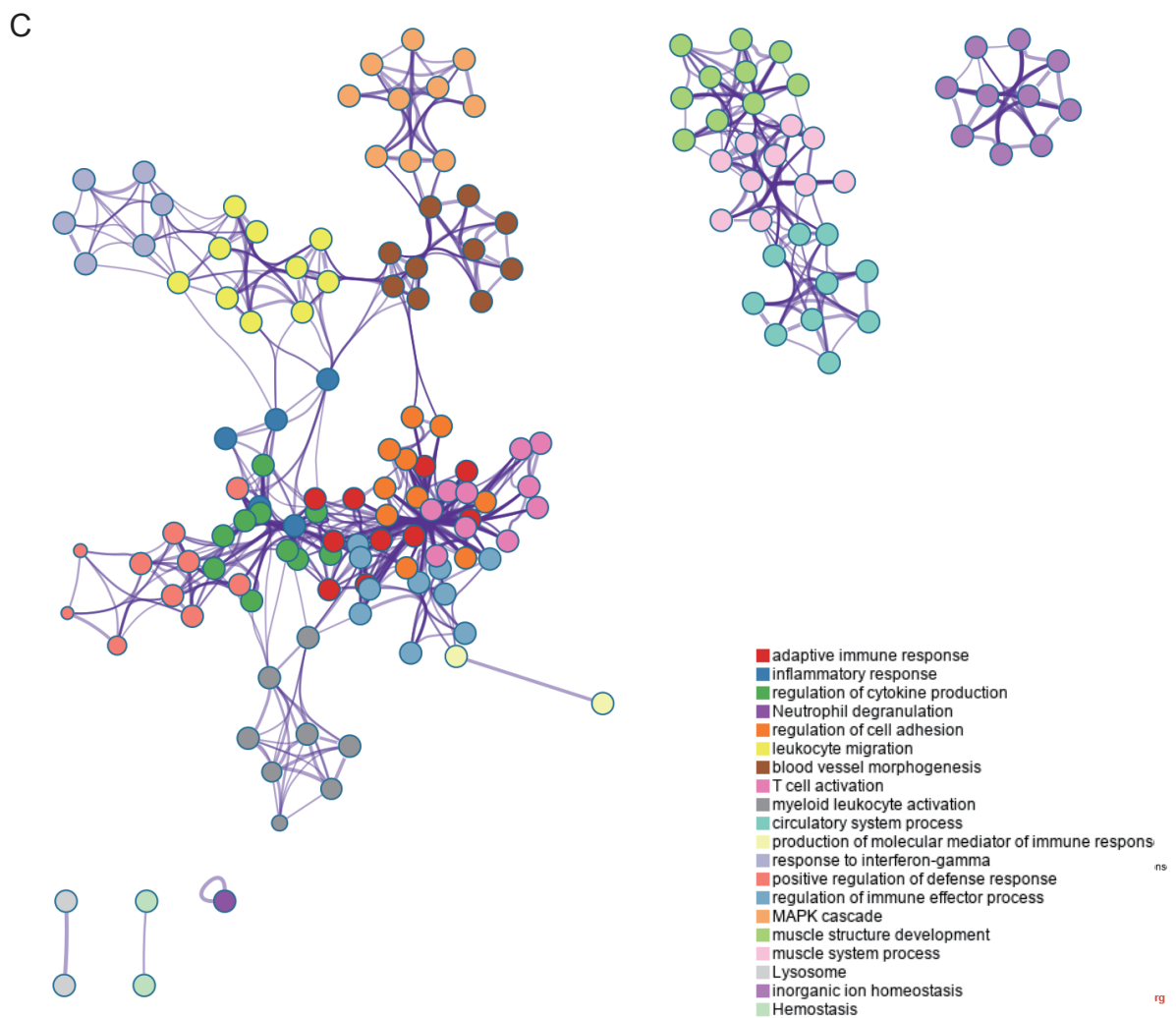

Supplement: Supplementary file 5 — Supporting Information [file CTM2-11-e590-s012.pdf]

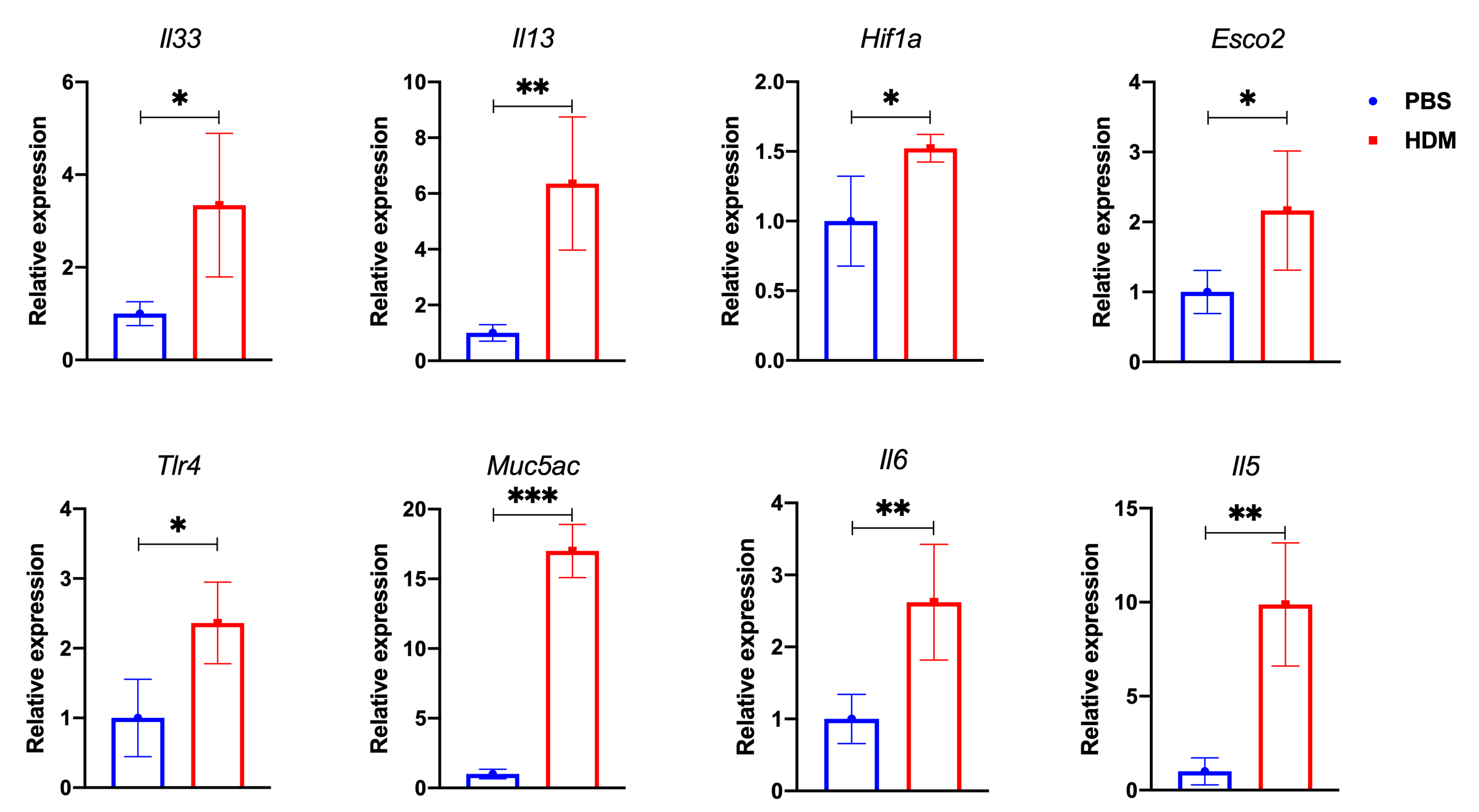

Supplement: Supplementary file 6 — Supporting Information [file CTM2-11-e590-s005.tiff]

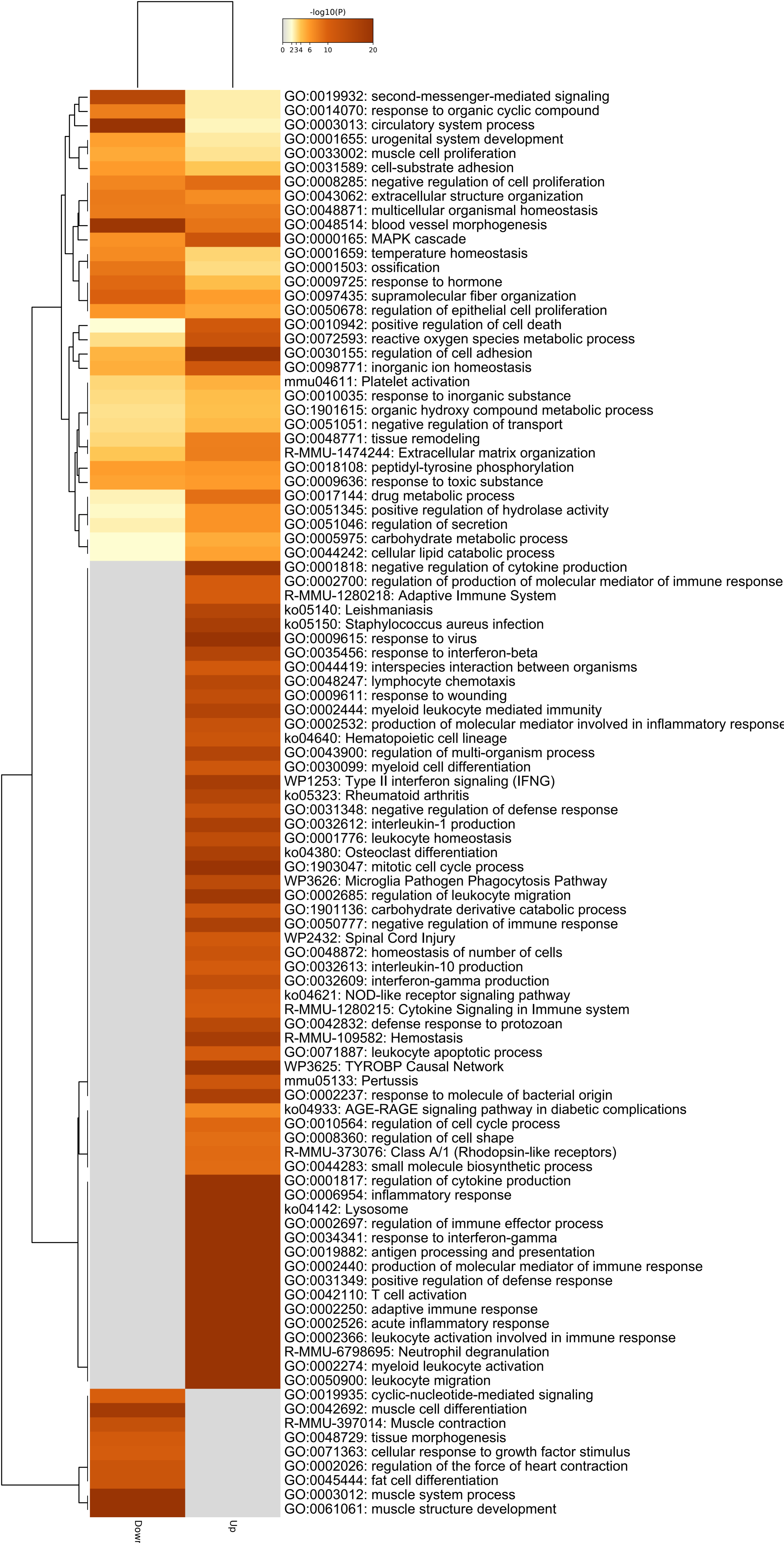

Supplement: Supplementary file 7 — Supporting Information [file CTM2-11-e590-s001.pdf]

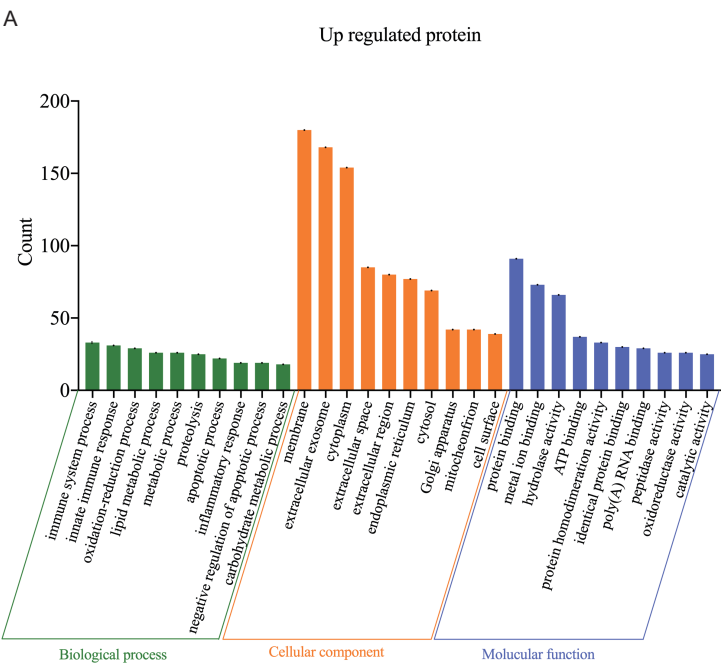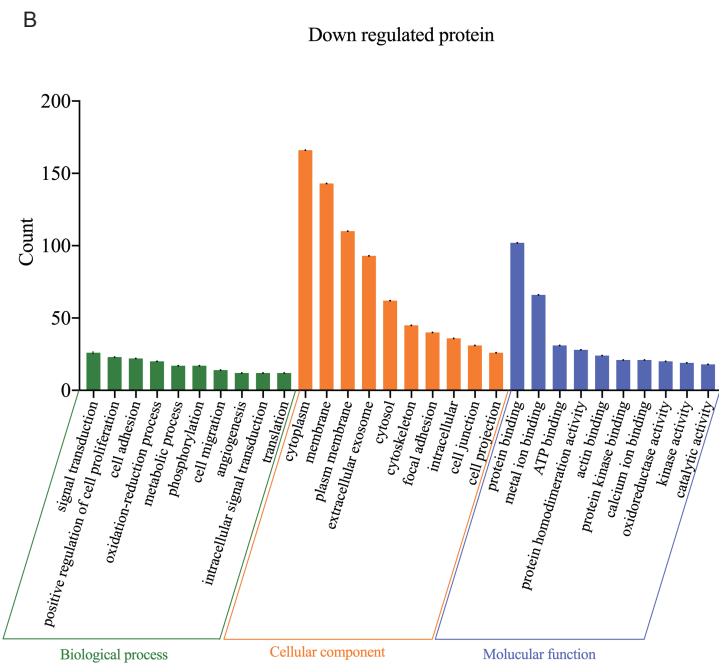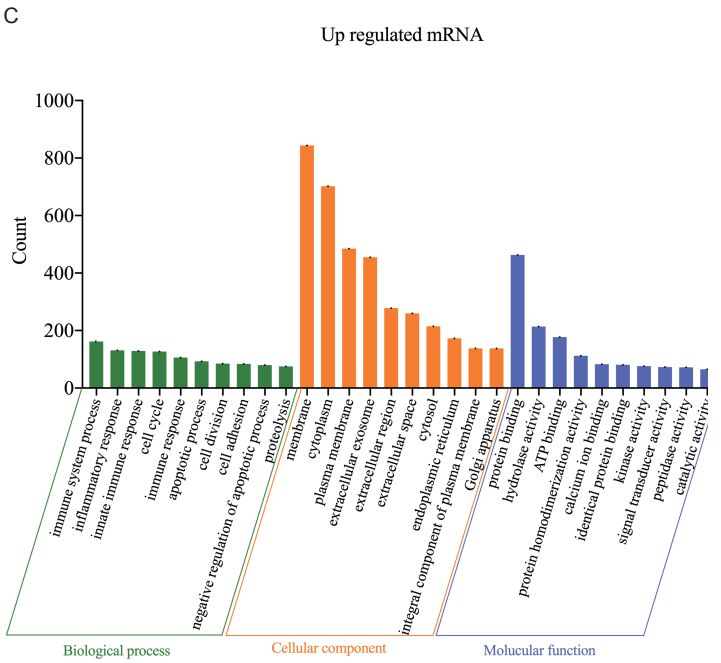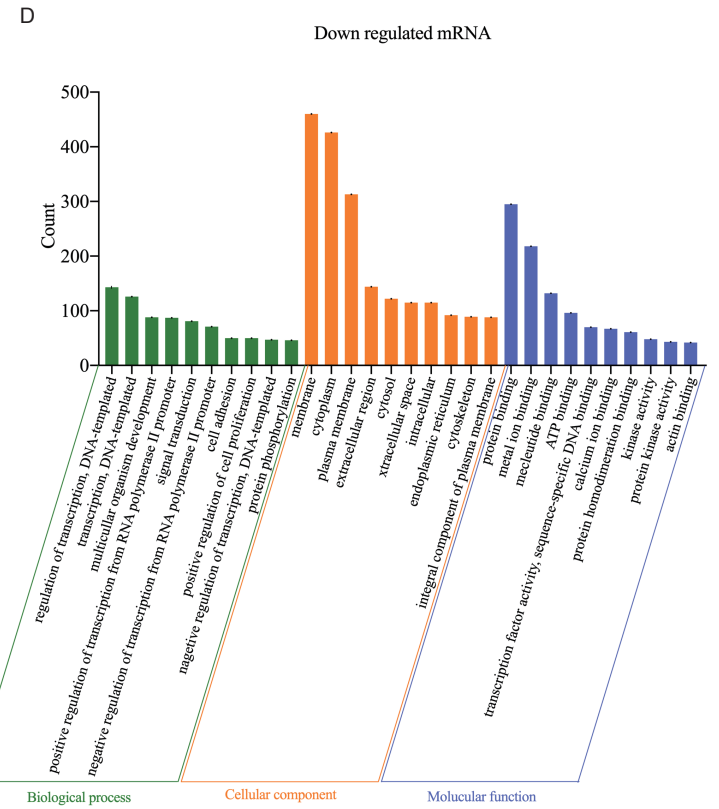

Supplement: Supplementary file 8 — Supporting Information [file CTM2-11-e590-s002.pdf]

A

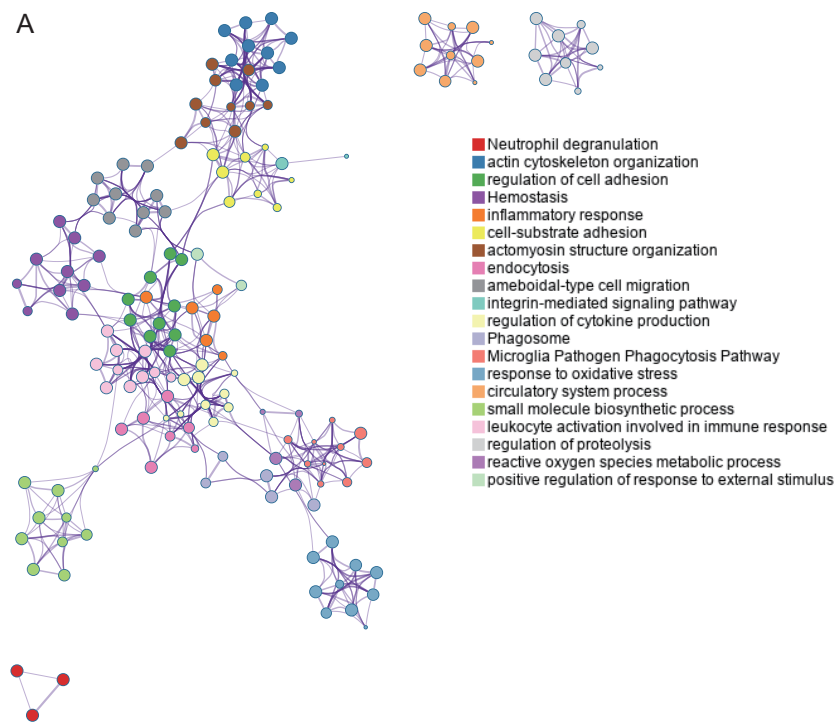

B

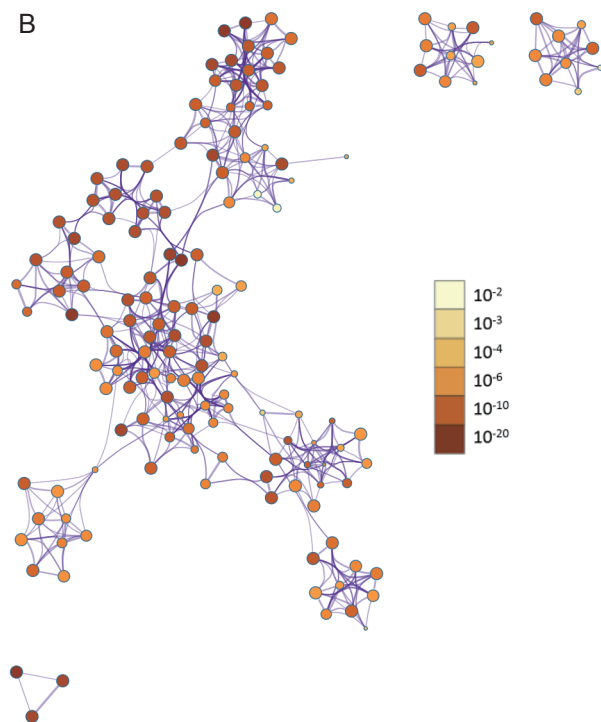

C

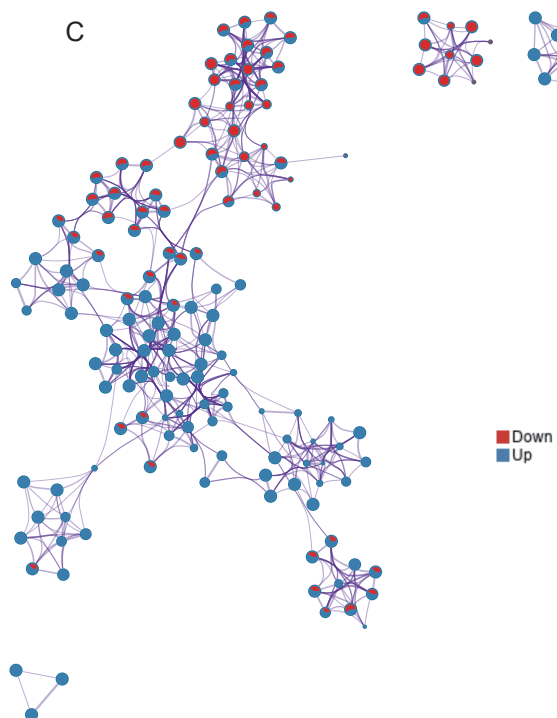

Supplement: Supplementary file 9 — Supporting Information [file CTM2-11-e590-s014.pdf]

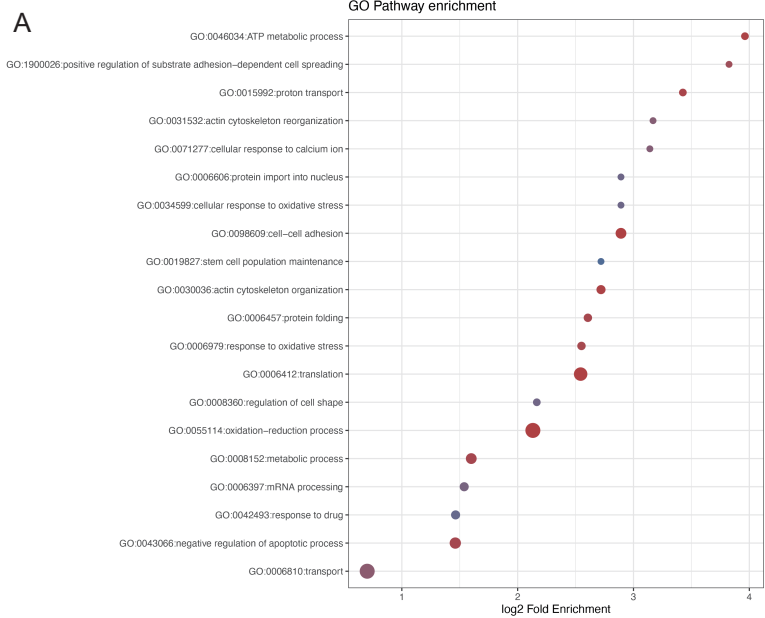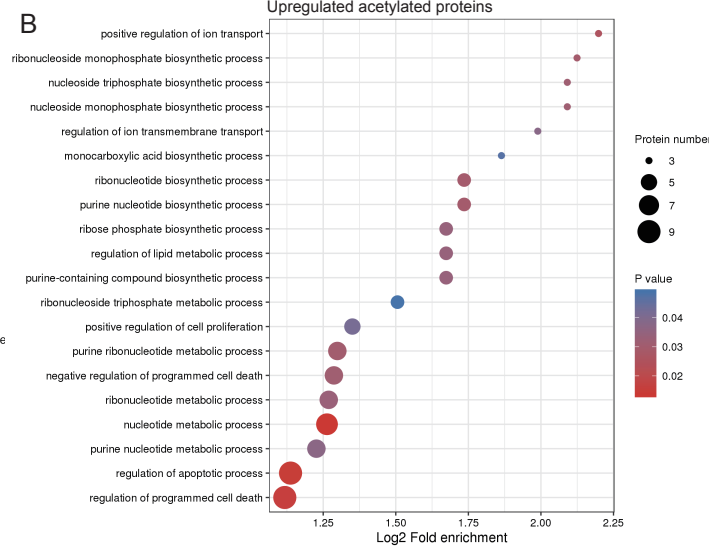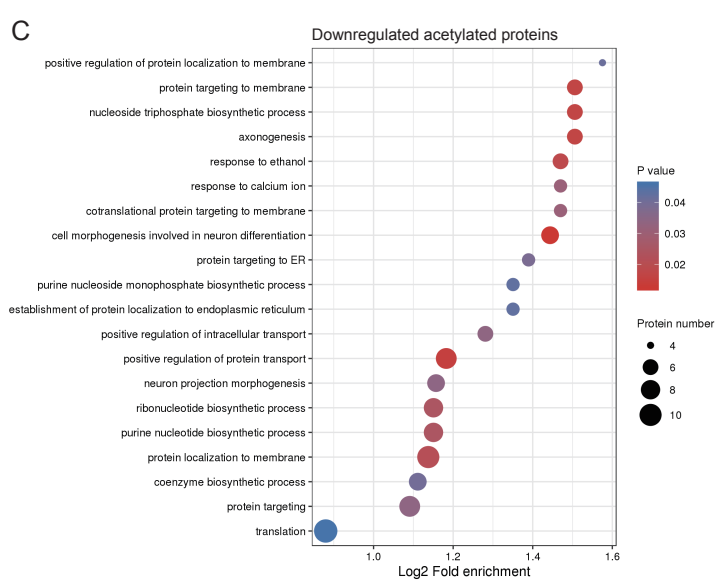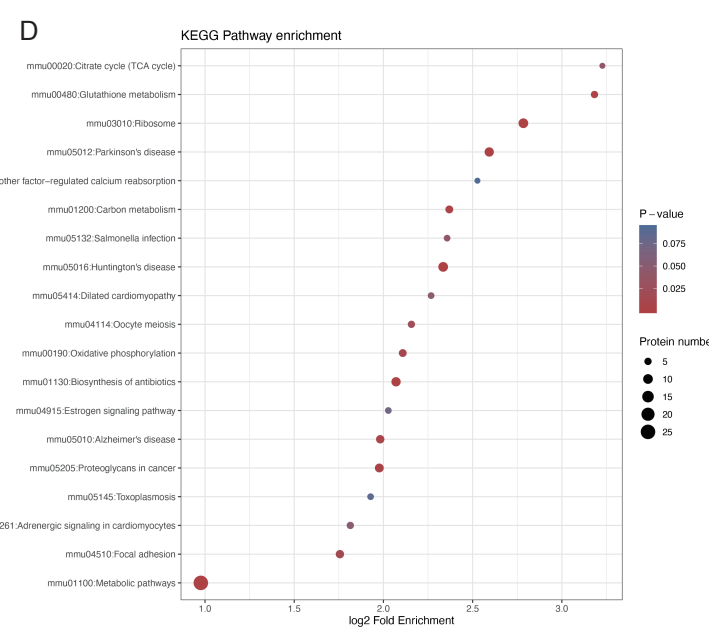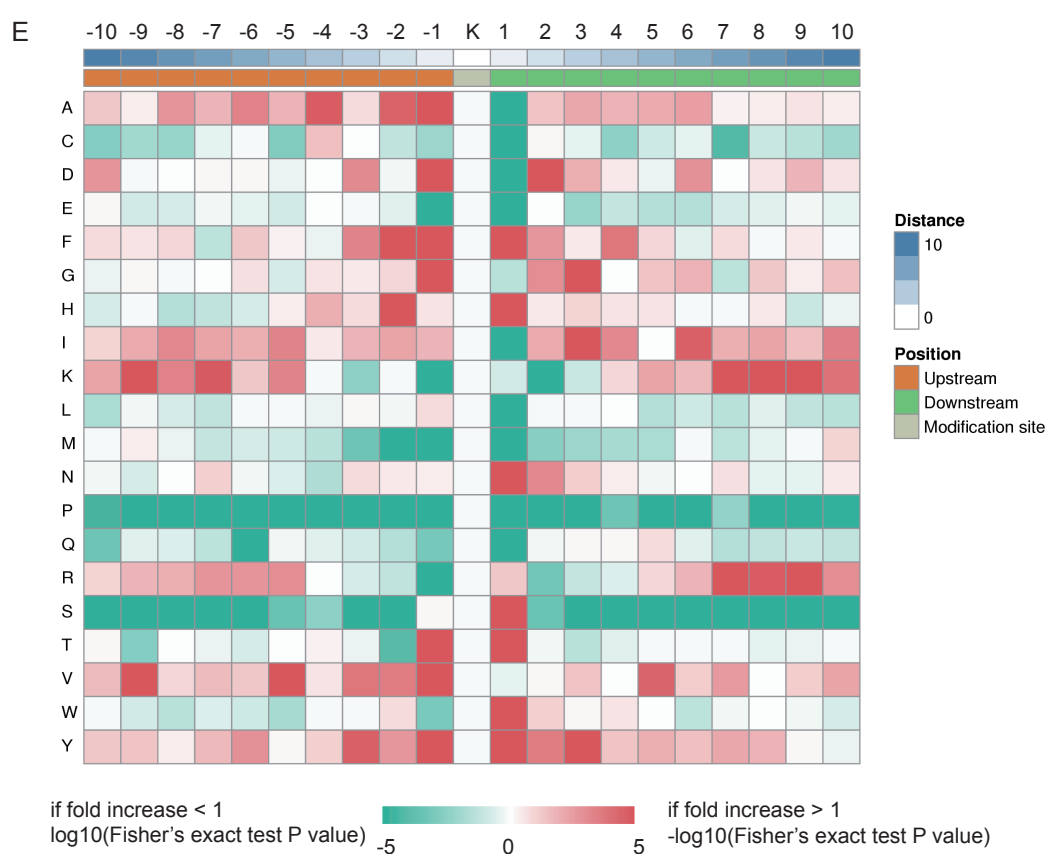

Supplement: Supplementary file 10 — Supporting Information [file CTM2-11-e590-s016.pdf]

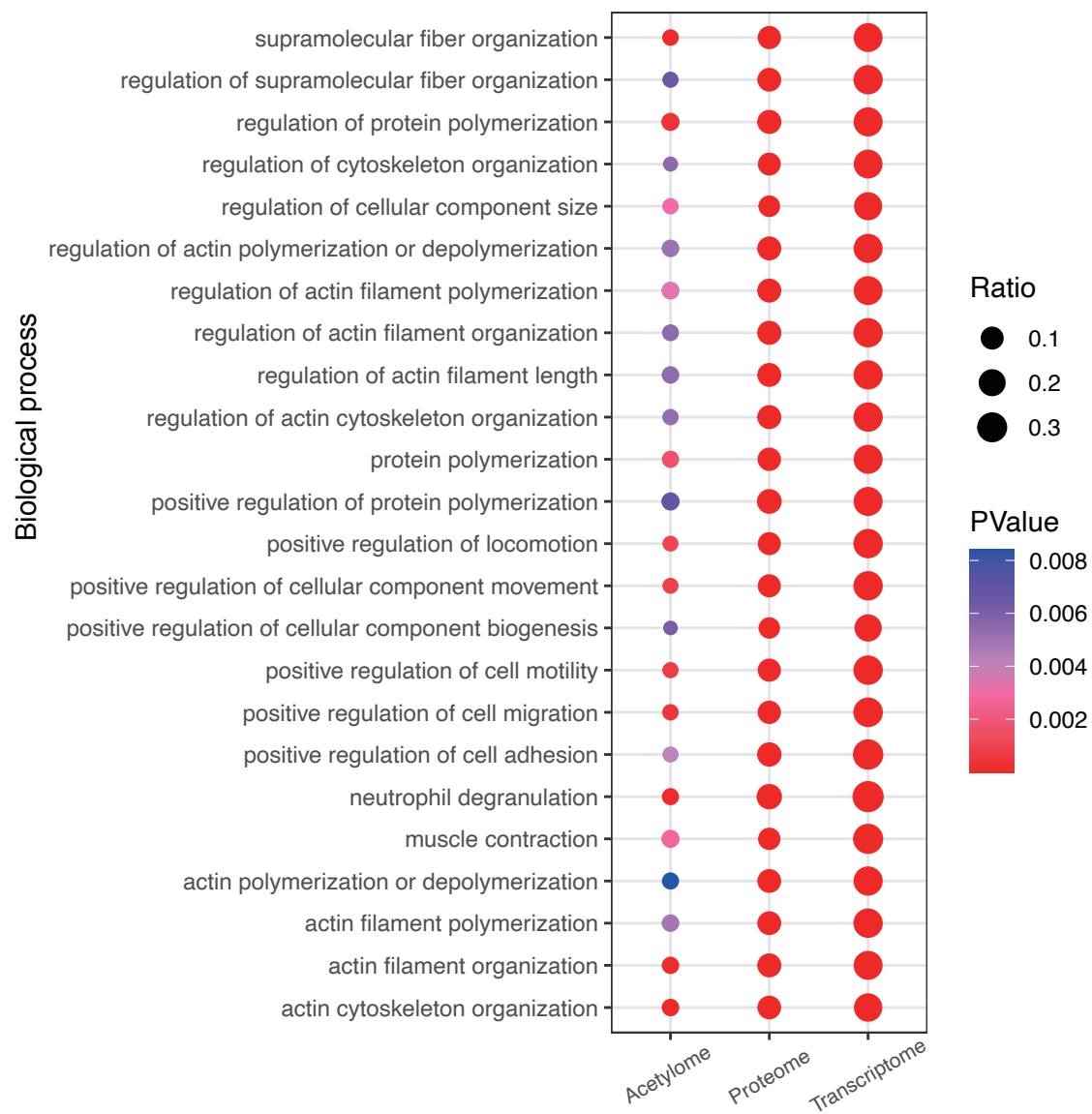

Supplement: Supplementary file 12 — Supporting Information [file CTM2-11-e590-s008.pdf]
