# Supplementary material for: Comparative analysis of global transcriptome, proteome and acetylome in house dust mite‐induced murine allergic asthma model
Source: Clin Transl Med. 2021 Nov 6;11(11):e590. doi: 10.1002/ctm2.590 (PMC8571946; doi:10.1002/ctm2.590)
Supplement: Supplementary file 11 — Supporting Information [file CTM2-11-e590-s007.pdf]

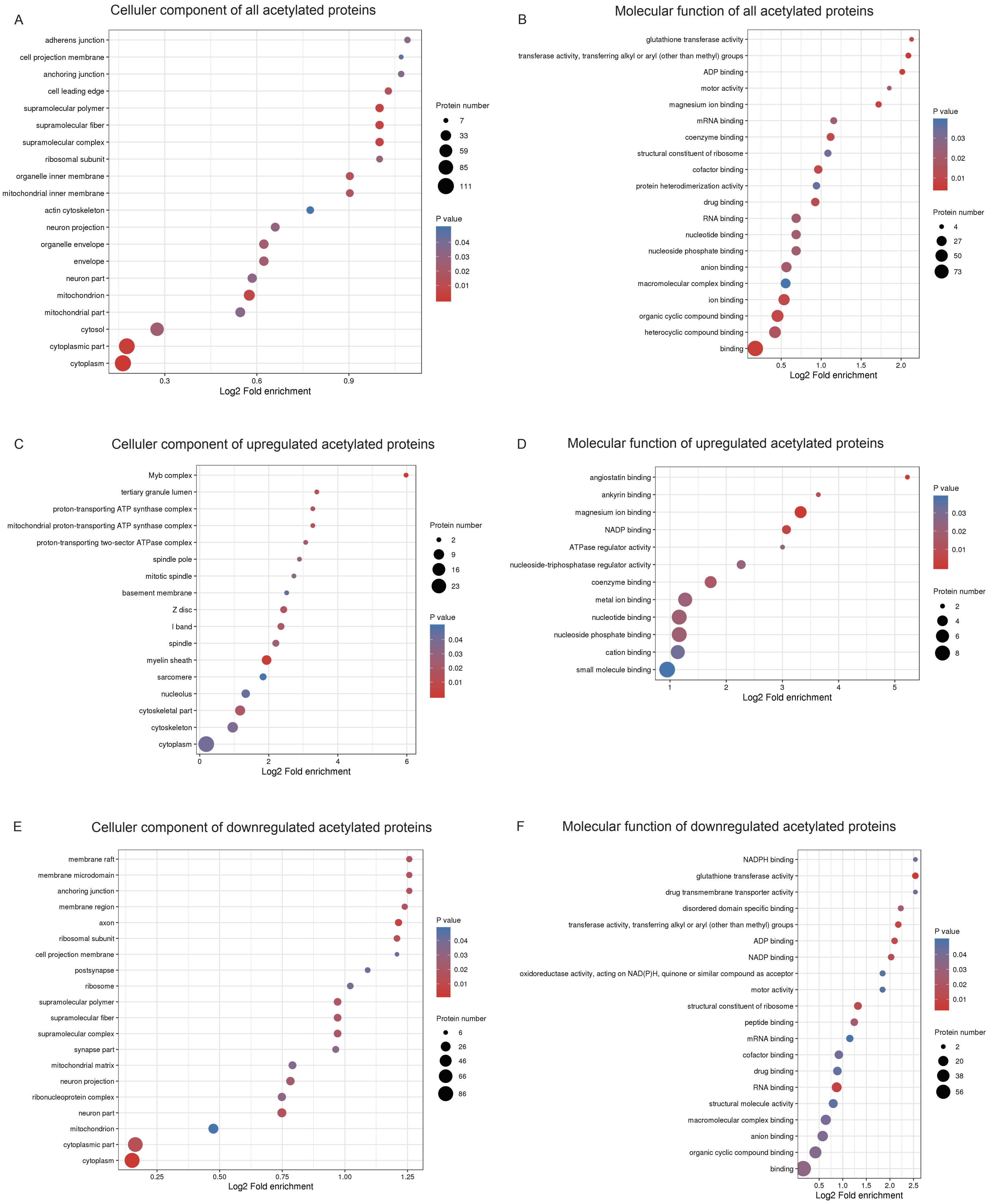
A
Celluler component of all acetylated proteins
adherens junction
cell projection membrane
anchoring junction
cell leading edge
supramolecular polymer
supramolecular fiber
supramolecular complex
ribosomal subunit
organelle inner membrane
mitochondrial inner membrane
actin cytoskeleton
neuron projection
organelle envelope
envelope
neuron part
mitochondrion
mitochondrial part
cytosol
cytoplasmic part
cytoplasm
0.3
0.6
0.9
Log2 Fold enrichment
Protein number
7
33
59
85
111
P value
0.04
0.03
0.02
0.01
B
Molecular function of all acetylated proteins
glutathione transferase activity
transferase activity, transferring alkyl or aryl (other than methyl) groups
ADP binding
motor activity
magnesium ion binding
mRNA binding
coenzyme binding
structural constituent of ribosome
cofactor binding
protein heterodimerization activity
drug binding
RNA binding
nucleotide binding
nucleoside phosphate binding
anion binding
macromolecular complex binding
ion binding
organic cyclic compound binding
heterocyclic compound binding
binding
0.5
1.0
1.5
2.0
Log2 Fold enrichment
P value
0.03
0.02
0.01
Protein number
4
27
50
73
C
Celluler component of upregulated acetylated proteins
Myb complex
tertiary granule lumen
proton-transporting ATP synthase complex
mitochondrial proton-transporting ATP synthase complex
proton-transporting two-sector ATPase complex
spindle pole
mitotic spindle
basement membrane
Z disc
I band
spindle
myelin sheath
sarcomere
nucleolus
cytoskeletal part
cytoskeleton
cytoplasm
0
2
4
6
Log2 Fold enrichment
Protein number
2
9
16
23
P value
0.04
0.03
0.02
0.01
D
Molecular function of upregulated acetylated proteins
angiostatin binding
ankyrin binding
magnesium ion binding
NADP binding
ATPase regulator activity
nucleoside-triphosphatase regulator activity
coenzyme binding
metal ion binding
nucleotide binding
nucleoside phosphate binding
cation binding
small molecule binding
1
2
3
4
5
Log2 Fold enrichment
P value
0.03
0.02
0.01
Protein number
2
4
6
8
E
Celluler component of downregulated acetylated proteins
membrane raft
membrane microdomain
anchoring junction
membrane region
axon
ribosomal subunit
cell projection membrane
postsynapse
ribosome
supramolecular polymer
supramolecular fiber
supramolecular complex
synapse part
mitochondrial matrix
neuron projection
ribonucleoprotein complex
neuron part
mitochondrion
cytoplasmic part
cytoplasm
0.25
0.50
0.75
1.00
1.25
Log2 Fold enrichment
P value
0.04
0.03
0.02
0.01
Protein number
6
26
46
66
86
F
Molecular function of downregulated acetylated proteins
NADPH binding
glutathione transferase activity
drug transmembrane transporter activity
disordered domain specific binding
transferase activity, transferring alkyl or aryl (other than methyl) groups
ADP binding
NADP binding
oxidoreductase activity, acting on NAD(P)H, quinone or similar compound as acceptor
motor activity
structural constituent of ribosome
peptide binding
mRNA binding
cofactor binding
drug binding
RNA binding
structural molecule activity
macromolecular complex binding
anion binding
organic cyclic compound binding
binding
0.5
1.0
1.5
2.0
2.5
Log2 Fold enrichment
P value
0.04
0.03
0.02
0.01
Protein number
2
20
38
56
