# Supplementary material for: Comparative analysis of global transcriptome, proteome and acetylome in house dust mite‐induced murine allergic asthma model
Source: Clin Transl Med. 2021 Nov 6;11(11):e590. doi: 10.1002/ctm2.590 (PMC8571946; doi:10.1002/ctm2.590)
Supplement: Supplementary file 15 — Supporting Information [file CTM2-11-e590-s017.docx]

**Table S3. Comparison of published transcriptome data.**

| Study | Year | Series accession | Strain | Environment | Modeling | Sacrifice |
| --- | --- | --- | --- | --- | --- | --- |
| Jeanne et al.^3^ | 2019 | GSE137324 | C57BL/6 mice | pathogen-free environment | HDM extract was resuspended in saline solution and administered intranasally at a dose of 50 mg to isoflurane-anesthetized mice 3 times per week for up to 18 consecutive weeks | 3 days after the last HDM administration |
| Li et al.^4^ | 2021 | GSE102211 | C57BL/6J mice | pathogen-free conditions | Each mouse was administered via the intratracheal route with 100 mg HDM extracts or PBS for three times on days 0, 7, and 14 | 3 days after the last HDM administration |
| Sabrina et al.^5^ | 2020 | GSE126610 | BALB/c mice | / | Mice were sensitized with 50 mL endotoxin-free normal saline containing 50 mg protein on days 0, 1, and 2. Animals were challenged daily with HDM extract (5 mg protein/50 mL, i.n.) on days 14, 15, 16, and 17 to elicit allergic sensitization. | 24 hours after the last HDM administration |
| Burleson et al.^6^ | 2019 | GSE124922 | B6/129S4 | / | On days 0 and 7, mice were sensitized intraperitoneally with 100 μg of HDM (representing 33 μg of protein) in 100 μL of PBS plus alum or an equivalent amount of PBS alone. On days 14, 19 and 21, mice were challenged intratracheally with 100 μg of HDM in 50 μL of PBS or PBS alone. | 2 days after the last HDM administration |
| Singhania et al.^7^ | 2019 | GSE119856 | C57BL/6J mice | pathogen-free conditions | Female C57BL/6J mice were sensitized with 10 mg HDM and 2 mg Imject Alum in 200 μl PBS or Alum alone as control by i.p. injections on days 0 and 14, followed by i.t. challenge with 10 mg HDM in 20 μl of PBS or PBS on days 21 and 24. | 24 hours after the last HDM treatment |
| Takashi et al.^8^ | 2017 | GSE100858 | C57BL/6 mice | pathogen-free conditions | mice were sensitized intratracheally with 50 μg HDM in 25 μl PBS twice at a 7-d interval. 7 d after the last sensitization, mice were challenged with 5 μg HDM for four consecutive days | 48 hours after the last HDM challenge |
| Kayla et al.^9^ | 2014 | GSE49047 | C57BL/6 mice | / | Mice were immunized with intratracheal instillation of 100 mg of HDM extract in 50 ml of PBS or 50 ml of PBS alone 3 times per week for 3 weeks | 24 hours after the last HDM challenge |
| Christopher et al.^10^ | 2020 | GSE116967 | BALB/c mice | / | HDM at 25 μg of protein in 35 μL of sterile saline intranasally five times a week for 2 weeks | 2 days after the last HDM administration |

Ref num: see the reference of the main text for details.
